# Pangenomic Analysis of Nucleo-Cytoplasmic Large DNA Viruses. I: The Phylogenetic Distribution of Conserved Oxygen-Dependent Enzymes Reveals a Capture-Gene Process

**DOI:** 10.1007/s00239-023-10126-z

**Published:** 2023-08-01

**Authors:** J. A. Campillo-Balderas, A. Lazcano, W. Cottom-Salas, R. Jácome, A. Becerra

**Affiliations:** 1 Facultad de Ciencias, UNAM, Cd. Universitaria, Apdo. Postal 70-407, 04510 Mexico City, DF Mexico; 2 El Colegio Nacional, Donceles 104, Centro Histórico, 06020 Mexico City, CP Mexico; 3 Escuela Nacional Preparatoria, Plantel 8 Miguel E. Schulz, Universidad Nacional Autónoma de México, Mexico City, Mexico

**Keywords:** Nucleo-cytoplasmic large DNA viruses, Gene recruitment, Origin and evolution of viruses, Pangenomic, Proterozoic great oxidation event

## Abstract

The Nucleo-Cytoplasmic Large DNA Viruses (NCLDVs) infect a wide range of eukaryotic species, including amoeba, algae, fish, amphibia, arthropods, birds, and mammals. This group of viruses has linear or circular double-stranded DNA genomes whose size spans approximately one order of magnitude, from 100 to 2500 kbp. The ultimate origin of this peculiar group of viruses remains an open issue. Some have argued that NCLDVs’ origin may lie in a bacteriophage ancestor that increased its genome size by subsequent recruitment of eukaryotic and bacterial genes. Others have suggested that NCLDVs families originated from cells that underwent an irreversible process of genome reduction. However, the hypothesis that a number of NCLDVs sequences have been recruited from the host genomes has been largely ignored. In the present work, we have performed pangenomic analyses of each of the seven known NCLDVs families. We show that these families' core- and shell genes have cellular homologs, supporting possible escaping-gene events as part of its evolution. Furthermore, the detection of sequences that belong to two protein families (small chain ribonucleotide reductase and Erv1/Air) and to one superfamily [2OG-Fe(II) oxygenases] that are for distribution in all NCLDVs core and shell clusters encoding for oxygen-dependent enzymes suggests that the highly conserved core these viruses originated after the Proterozoic Great Oxidation Event that transformed the terrestrial atmosphere 2.4–2.3 Ga ago.

**Supplementary Information:**

The online version contains supplementary material available at 10.1007/s00239-023-10126-z.

## Introduction

The Nucleo-Cytoplasmic Large DNA Viruses (NCLDVs) infect eukaryotic hosts including amoeba, algae, fish, amphibia, arthropods, birds, and mammals. These viruses have linear or circular double-stranded DNA genomes whose size spans approximately one order of magnitude, from 100 to 2500 kbp. Seven taxonomic families are currently recognized by the International Committee on Taxonomy of Viruses as members of the NCLDVs (ICTV 2020). These families are the *Ascoviridae, Asfarviridae, Iridoviridae, Marseilleviridae, Mimiviridae, Phycodnaviridae*, and *Poxviridae* (Tidona and Darai 2011). These viral families have also been grouped into the Phylum *Nucleocytoviricota* because they either synthesize their DNA exclusively in the cytoplasm, or have a first-stage replication and early transcription in the host nucleus and a late transcription in its cytoplasm (Chinchar and Hyatt 2008; Koonin and Yutin 2010; Asgari et al. 2017).

Forty-seven core proteins have been identified in NCLDVs. However, only ten of them (A2L-like transcription factor, A32-like-packaging ATPase, D5-like helicase primase, elongation subunit of family B DNA polymerase, helicase II, the large subunit of mRNA capping enzyme, myristoylated envelope protein, the small subunit of ribonucleotide reductase, and the RNA polymerase α- and β- subunits) have been proposed as phylogenetic markers. They indicate that NCLDVs are a monophyletic group (Yutin et al. 2009). This suggests that the common ancestor of NCLDVs may have been endowed with an icosahedral capsid, DNA replication, and transcription genes, proteins involved in virion morphogenesis and perhaps, also inhibitors of apoptosis (Iyer et al. 2001). This hypothetical ancestral viral population may have also undergone gene loss of essential genes and possible non-orthologous host gene displacements in its current descendents (Iyer et al. 2001; Koonin and Yutin 2010, 2012; Yutin and Koonin 2012). It has also been suggested that this hypothetical common ancestor probably evolved from a bacteriophage, and that subsequent recruitment of eukaryotic and bacterial genes led to an early radiation of the NCLDVs that was associated with the origin of eukaryotes (Koonin and Yutin 2010). In addition, it has been proposed that the horizontal gene transfer of bacteriophage, archaeal, and plasmid selfish genes was, and continues to be, an important process in the NCLDVs emergence and evolution (Koonin et al. 2006; Moreira and Brochier-Armanet 2008). These ancestral NCLDVs might have encoded a minimal set of genes, but recurring genetic expansion-reduction cycles, i.e., a “genomic accordion” led to a diversity of large viral genomes (Filée 2013). Some authors have assumed that NCLDVs and other eukaryotic dsDNA viruses have independently originated from Tectiviridae phages with evolutionary intermediate mobile genetic elements (a.k.a polintons and polintoviruses) (Koonin et al. 2015) with a highly genetic contribution of bacteria (Bäckström et al. 2019).

Others have argued that NCLDVs originated from an irreversible process of cellular genome reduction comparable to the ones that have led to obligate intracellular symbionts, in which ribosomal genes were lost, but some other translational and transcriptional genes were conserved (Tamames et al. 2007; Claverie and Abergel 2013). Claverie and Abergel (2013) have argued that “[this scenario] might represent an evolutionary link between the emergence of the cell nucleus and the origin of the large DNA viruses.” Finally, based on the phyletic patterns of putative orthologous genes encoding ribonucleotide reductase, thymidylate synthase, B-family DNA polymerase, topoisomerase II-A proliferating cell nuclear antigen, flap endonuclease, RNA polymerase, transcription factor TFIIB, and some aminoacyl-tRNA synthetases, it has also been suggested that NCLDVs emerged directly from the root of the universal tree of life as a fourth major domain in addition to the Bacteria, Archaea, and the Eukarya (Boyer et al. 2010; Legendre et al. 2012; Woyke and Rubin 2014).

In the present work, we report the results of a pangenomic analysis of the proteomic repertoire, i.e., core, shell, and cloud of NCLDVs homologous protein clusters. We propose that since several core proteins present in each of the seven NCLDVs families have corresponding cellular homologs, a mechanism of host-escaping genes may be the most plausible explanation for the origin of this viral group. The presence of 13 strictly O_2_-dependent enzymes and, more specifically, small subunit ribonucleotide reductase type Ia, Erv1/Alr, and 2OG-Fe(II) oxygenases, at the core and shell of different NCLDVs families, suggests that a number of highly conserved genes for the replication cycle of these viruses were acquired from their eukaryotic hosts following the Great Oxidation Event (GOE) that changed the terrestrial environment over 2.4–2.3 billion years ago.

## Methodology

### Retrieval and Pangenomic Analysis of NCLDVs Genomes

All viral and cellular complete RefSeq proteomes were downloaded from the NCBI GenBank (https://www.ncbi.nlm.nih.gov/genome/viruses/ available as of January 2022) and the 2017 KEGG database, respectively. The viral proteome files were formatted and classified according to the seven currently recognized NCLDVs families (A*scoviridae, Asfarviridae, Iridoviridae, Marseilleviridae, Mimiviridae, Phycodnaviridae*, and *Poxviridae*) using Perl scripts. NLCDV proteomes currently not classified into a viral family or with a partial sequence were excluded.

The GET_HOMOLOGUES software for pangenomic analysis was used to obtain the homologous protein clusters of all NCLDV families according to the command instructions of the manual (Contreras-Moreira and Vinuesa 2013). Given the divergent nature of viral sequences, we have empirically adapted the concept of pangenome (Medini et al. 2005; Tettelin et al. 2008) to define a core that includes homologous proteins shared by at least 95% (core + softcore) of all species of a viral family, a shell that covers partially shared proteins (< 95%), and a cloud that comprises all the remaining proteins present in one or two viral species. Viral families with less than three RefSeq proteomes were also discarded from pangenomic comparisons. The bias in the diversity of the size and nature of the proteomes for the classification by families of these viruses was, thus, considered.

Clusterization of homologous proteins of each NCDLV family was performed using the smallest RefSeq viral proteome as a query. The paired search was done by the combination of BLASTP (Altschul et al. 1990), HMMER (Eddy 2009), and COGTriangles (Kristensen et al. 2010) algorithms with an alignment query coverage of 75% and a Evalue < 10E-05. Proteins with no other viral species homologs in the same family (orphans) were separated. To identify the corresponding conserved domains, a search was conducted in the Pfam database Version 28.0 (Finn et al. 2008).

The estimation of the pangenomic repertoire size (core, shell, and cloud) was calculated. The data were extrapolated by fitting the Tettelin exponential decaying function model (Tettelin et al. 2005). Once the protein presence–absence matrix of each viral family was generated, all these data were counted and classified into the pangenomic compartments, and then plotted by calling R functions (https://www.r-project.org) at GET_HOMOLOGUES. The functional classification of pangenomic orthologous clusters, the information of Pfam, GenBank, Uniprot, Gene Ontology, and KEGG databases was used through Pfam and GenBank accession numbers once identified by GET_HOMOLOGUES. This classification system is based on protein sequence homology of complete prokaryotic and eukaryotic genomes (Tatusov 1997) and was used to categorize the NCLDVs orthologous clusters according to the predicted function using Pfam database or cited references. These categories, identified by one letter, belong to four general functions: (i) information storage and processing (A, B, J, K, L); (ii) cellular processes and signaling (D, M, N, O, T, U, V, W, Y, Z); (iii) metabolism (C, E, F, G, H, I, P, Q); and (iv) poorly characterized functions (R, S). In this work, we propose two additional viral categories for this classification system: (v) miscellaneous functions (X), which include orthologous clusters that partake in many unrelated functions (genetic information, cellular processes, and metabolism); and (vi) capsid-related functions (Vc), which are viral orthologous clusters that have no cellular counterpart at the Pfam database. NCLDVs COGs were also classified according to their relative frequency at the Core, Shell, and Cloud repertoires. All these genetic, cellular, and metabolic functions were classified according to the database of Clusters of Orthologous Groups of proteins (COGs, https://ftp.ncbi.nih.gov/pub/wolf/COGs/COG0303/fun.txt) (Tatusov 1997). Clusters without identifiers in all databases were classified as poorly characterized functions (COGs R and S). All those NCLDVs orthologous clusters with a Pfam identifier were used to determine their distribution in other viral groups, as well in Bacteria, Archaea, and Eukarya in the database. All of these orthologous clusters were counted according to their corresponding viral and domain distribution, NCLDVs families, and COG functions. The values were logarithmically normalized and plotted with a heatmap library by R software.

### NCLDV Oxygen-Dependent Enzyme Database and Phylogenies

From the NCLDV pangenomic database described above, all O_2_-dependent enzyme information and sequences were extracted in order to build a new database. The NCLDVs oxygen-dependent enzyme clusters with Pfam identifiers were selected to search through the KEGG database for distant homologous proteins in other viral groups, as well as in Bacteria, Archaea, and Eukarya. MAFFT software (Katoh et al. 2002) was used to construct multiple alignments with NCLDVs cluster sequences, except orphans. HMMER was used to perform a profile HMM with more than two NCLDVs sequences, while jackhammer was used to more accurately detect cellular homology in NCDLV orphans (Madera and Gough 2002). Both software packages were used with cut-off values of E < 10E-3. Redundant homologous sequences with a similarity threshold greater than 80% detected by the CD-HIT software (Fu et al. 2012) were discarded. All cell and virus homologous sequences were counted for each of the taxonomic groups (according to viral families and cellular phyla, kingdoms, and domains) with Bash and Awk scripts. All redundant sequences were removed by an Awk script.

MAFFT was used with default parameters to construct the multialignment of viral proteins and their cellular homologs. To remove the spurious sequences and poorly aligned regions of the multiple alignment, TrimAL software (Capella-Gutiérrez et al. 2009) was handled with default parameters. Maximum-likelihood phylogenies from sequence alignments were estimated using the best-fit model automatically selected by ModelFinder and ultrafast bootstrap with 1000 replicates implemented in IQ-TREE version 2.2.2.6 (Nguyen et al. 2015). The root was inferred without an outgroup by *rootstrap* using the most general amino acid non-reversible model (Naser-Khdour et al. 2022). Alternatively, a statistical test of the root was applied by comparing the log likelihoods of the trees rooted on every branch of the ML tree (tree topology test, –root test). The rooting position on branches in the test ID = 1 agrees with the highest rootstrap score. To visualize and edit the phylogenetic trees, the online iToL platform (Letunic and Bork 2016) was used.

## Results

### Database of NCLDVs Proteomes

Proteomic records of 136 species of *Ascoviridae* (6), *Asfarviridae* (8), *Iridoviridae* (26), *Marseilleviridae* (8), *Mimiviridae* (6), *Phycodnaviridae* (31), and *Poxviridae* (51) were used for the pangenomic analyses. Although the proteomes of *Pandoravirus* and *Pithovirus* were used for comparisons, they were not considered in pangenomic analyses due to the lack of more than three proteomes (Fig. S1). The relationship between the viral proteome size and the host classification is shown in Fig. S1. NCLDVs which infect invertebrates and vertebrates typically have less than 200 proteins, whereas those infecting protists typically have an expanded genome encoding for over 500 proteins. As shown in Table 1, the *Phycodnaviridae* family was the most affected by proteomic filtration (from 23 to 6 proteomes), due to their proteome size heterogeneity (from 150 to 886 proteins), which might affect the clustering process by GET-HOMOLOGUES. This viral family also infects a wide range of protist hosts.

**Table 1:** Homologous protein clusters of NCLDV

Viral family	Number of proteomes	Hosts	Number of proteins by proteome	Total number of proteins by family	Number of clusters					
					Core		Shell		Cloud	
					Orthologs	In-paralogs	Orthologs	In-paralogs	Orthologs	In-paralogs
Ascoviridae	6	Insects: lepidoptera and hymenoptera	119–194	970	33	1	112	3	353	7
Asfarviridae	8	Mammals: pigs, bushpigs, and warthogs Vector: argasid ticks	152–164	1279	138	2	29	4	36	0
Iridoviridae	26	Arthropods: insects and crustaceans Vertebrates: amphibians and fish	95–468	4105	10	0	318	10	1415	25
Marseilleviridae	8	Protists: *Acanthamoeba*	296–491	3468	216	17	236	11	708	8
Mimiviridae	6	Protists: *Cafeteria* and *Acanthamoeba*	544–1217	5742	307	14	329	10	2147	40
Phycodnaviridae	31	Protists (algae): Chlorophyta, Haptophyta, and Stramenopiles	150–886	11568	6	4	655	58	5192	201
Poxviridae	51	Arthropods; hexapoda; insecta; Pterygota Vertebrates: fish, birds, reptiles, mammals	120–334	10202	28	1	531	63	1901	146
			Total of proteins or clusters	37334	738	39	2210	159	11752	427

### Pangenomic Analysis of NCLDVs Proteomes

**Fig. 1:**
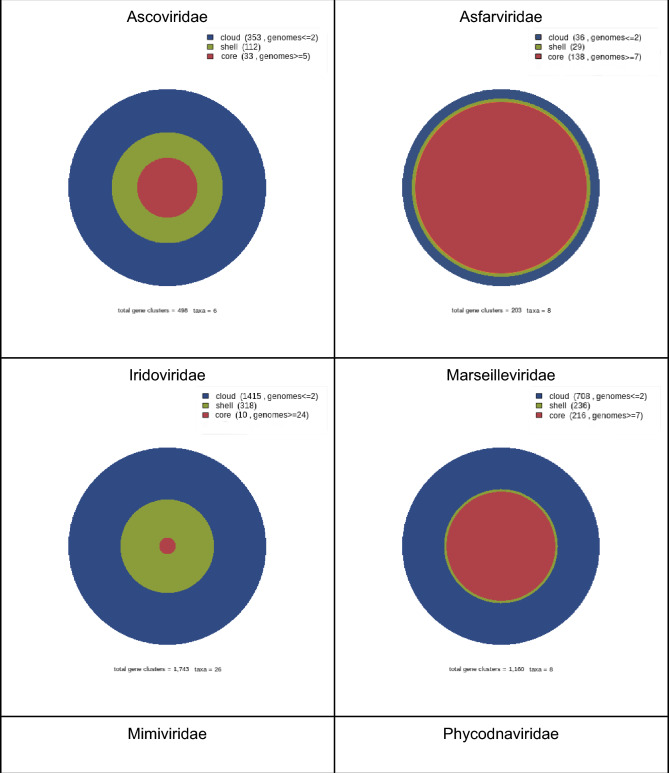

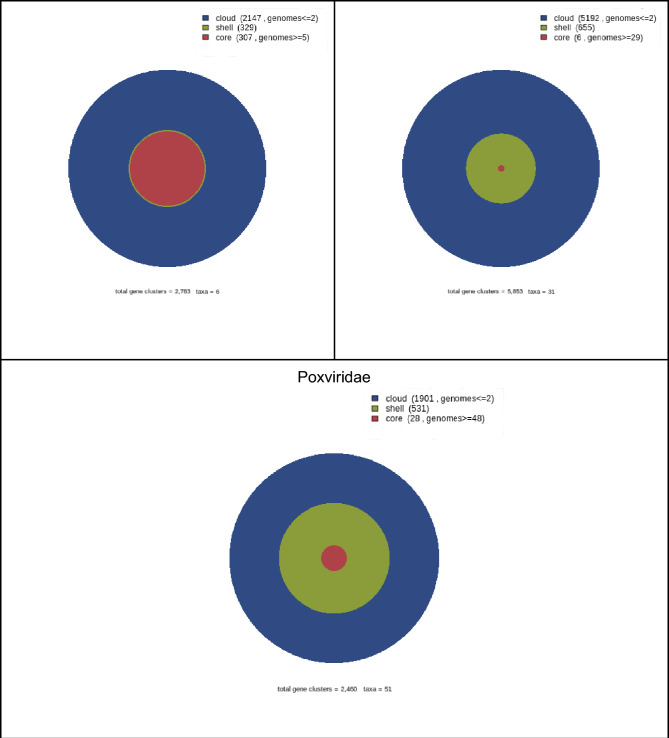
Pangenome of NCLDVs families. Orthologous clusters are plotted as relative frequency partitions of the pangenomic matrix into Core (red), Shell (green), and Cloud (blue) compartments (Color figure online)

A pangenomic analysis of more than 18,000 proteins from reference proteomes of *Asfarviridae, Ascoviridae, Iridoviridae, Marseilleviridae, Mimiviridae, Phycodnaviridae*, and *Poxviridae* is shown in Table1. Estimates of proteins included in the core, shell, and cloud of each viral family, were extrapolated by fitting the Tettelin exponential decaying function to the data as shown in Fig. 1.

### Functional Classification of the NCLDVs Pangenome

**Fig. 2:**
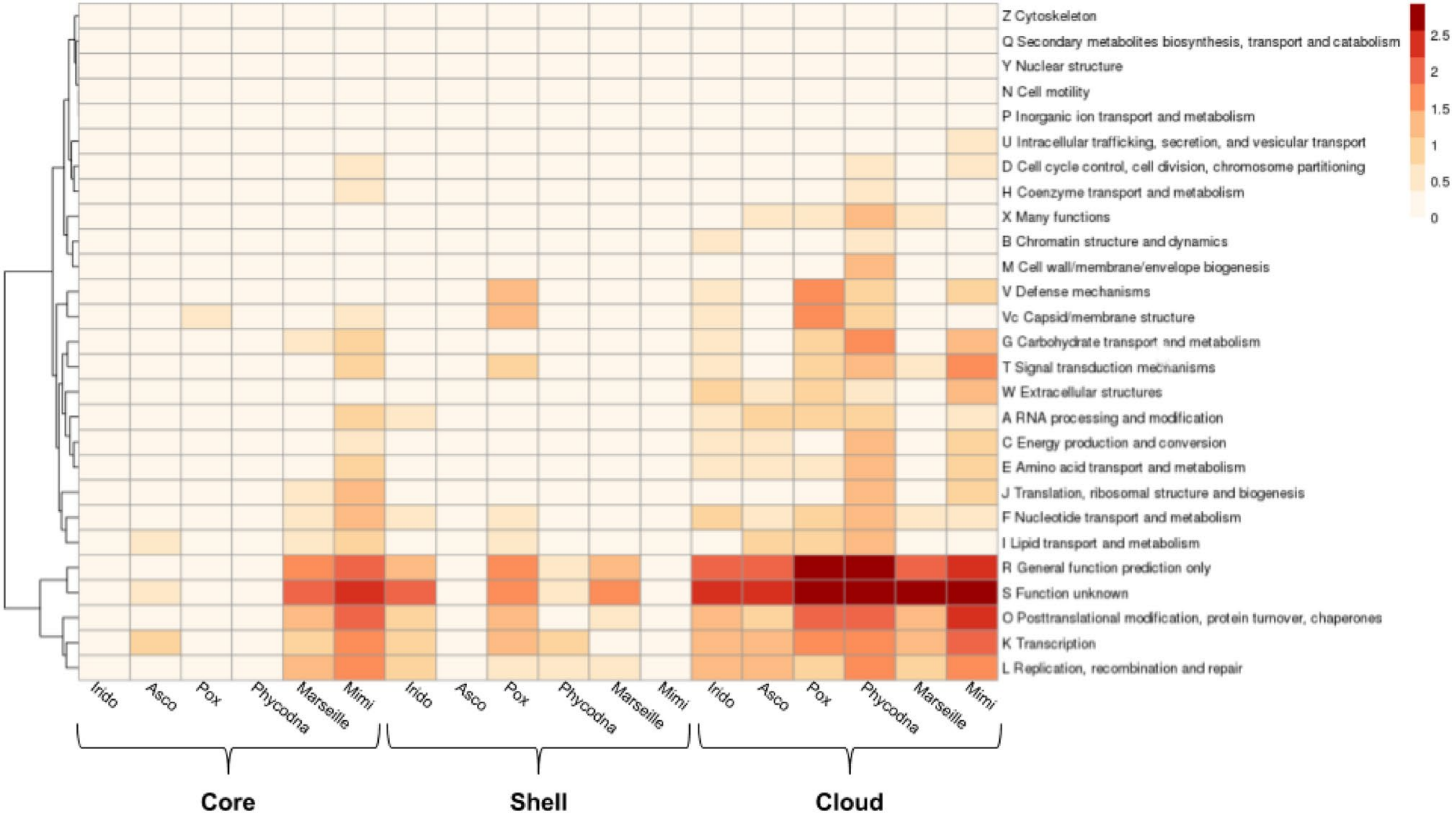
Functions of viral pangenome clusters. At the Y-axis, the functions are divided according to COGs described by (Tatusov 1997) with 10-base logarithm-normalized values. These functions intervene in the information storage and processing (A, B, J, K, L), cellular processes and signaling (D, M, N, O, T, U, V, W, Y, Z), metabolism (C, E, F, G, H, I, P, Q), and poorly characterized functions (R, S). Other categories were added to this work: miscellaneous (X) and capsid-related functions (Vc). At the X-axis, the functions are grouped according to the frequency of the orthologs in the Core, Shell, and Cloud clusters in each of the NCLDVs families (*Iridoviridae, Ascoviridae, Poxviridae, Phycodnaviridae, Marseilleviridae*, and *Mimiviridae*). According to the frequency (shades of red), orthologous clusters are divided into three categories: null or little abundance (mainly cellular processes such as cytoskeleton or coenzyme biosynthesis), moderately abundant (mainly viral processes such as capsid formation, signal transduction as tyrosine kinase receptors and nucleotide biosynthesis such as ribonucleotide reductase), and highly abundant (unknown functions, transcription such as RNApol and transcription and apoptosis factors such as repeated ankyrin and replication domains such as DNApol). *Ascoviridae* and *Mimiviridae* have no Shell due to only three genomes in the sample

As shown in Fig. 2, orthologs belonging to core-, shell-, and cloud clusters have been classified according to the Cluster of Orthologous Groups (COGs) into three categories, from the lowest to the highest frequency of proteins. The null or less abundant category (at the top of the heatmap) encompasses orthologous clusters that mediate cellular processes like cell cycle (e.g., cyclins, COG D) or membrane dynamics (e.g., ABC transporters, COG M), metabolic processes like coenzyme transport (e.g., N-acetyl transferase, COG H), and genetic information processes like chromatin remodeling (e.g., histones, COG B). Other orthologous clusters like ankyrin and leucine repetitions, and ATPases found in this category are overrepresented in NCLDVs with the largest genomes (*Poxviridae, Phycodnaviridae, Marseilleviridae,* and *Mimiviridae*).

The moderately abundant category located at the middle of the heatmap comprises orthologous clusters that partake in viral functions such as capsid/membrane structure (e.g., Poxvirus P4B major core protein, lipid membrane protein), and cellular processes, including defense mechanisms (e.g., chemokines, COG V), signal transduction (e.g., tyrosine/serine kinases, COG T), and extracellular structures (e.g., collagene, COG W). In addition, this same category includes orthologous clusters that have metabolic functions such as metabolism and transport of amino acids (e.g., glutamine transferase, COG E), nucleotides (e.g., dihydrofolate reductase, COG F), carbohydrates (e.g., glycosyltransferase, COG G), lipids (triacylglycerol lipase, COG I), and energy production (e.g., cytochrome P450, COG C). This moderately abundant category includes clusters with sequences associated with genetic information, such as RNA processing (RNA helicases, ribonuclease III domain; COG A) and translation (tRNA synthetases, translation initiation factor 4E; COG J).

The most abundant category includes orthologous clusters with no assigned function and is located at the bottom of the heatmap. This type of orthologs represents up to 70% of the *Marseilleviridae-* and *Mimiviridae-* pangenomic core (Raoult 2004; Boyer et al. 2010); however, as of today, we ignore their actual function, given the many unknowns in our understanding of NCLDVs. Some of them may be related to capsid assembly (Sobhy et al. 2015), whereas the molecular signatures of others suggest that they are involved in genetic information functions (COG L), including transcription and translation factors, as well as cellular processes such as signal transduction and apoptosis (COG O).

**Fig. 3:**
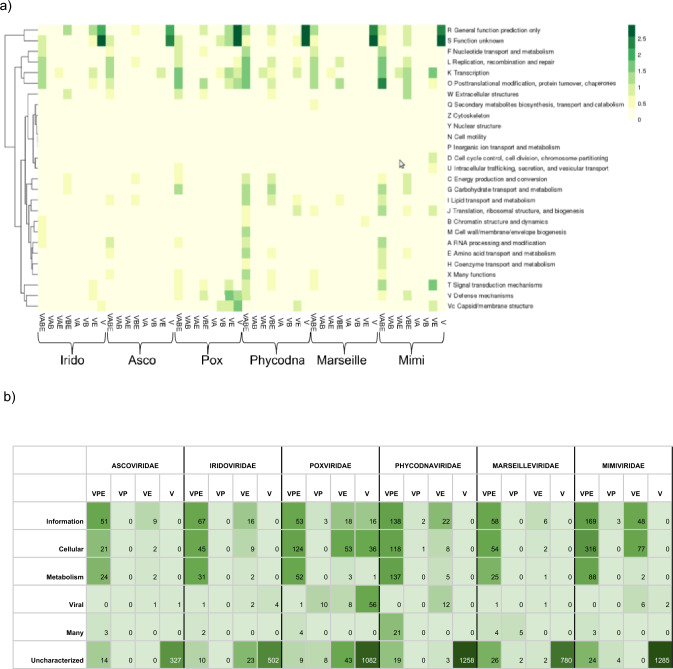
Comparison of function and domain distribution of the NCLDVs orthologous clusters. **a** At the Y-axis, COGs nomenclature is the same as shown in Fig. 2. At the X-axis, the frequency of the orthologous clusters are shown in NCLDVs and other viruses (V), in Archaea (A), in Bacteria (B), and in Eukarya (E). **b** Number of homologous protein clusters by megaviral family, by hosts (*V* Virus, *P* Procaryote, *E* Eukaryote), by general function (information, cellular, metabolism, viral, many, and uncharacterized). Most of them belong to viral clusters with no cellular homologs, but there are also many clusters with cellular homologs distributed into all VPE and VE

**Fig. 4:**
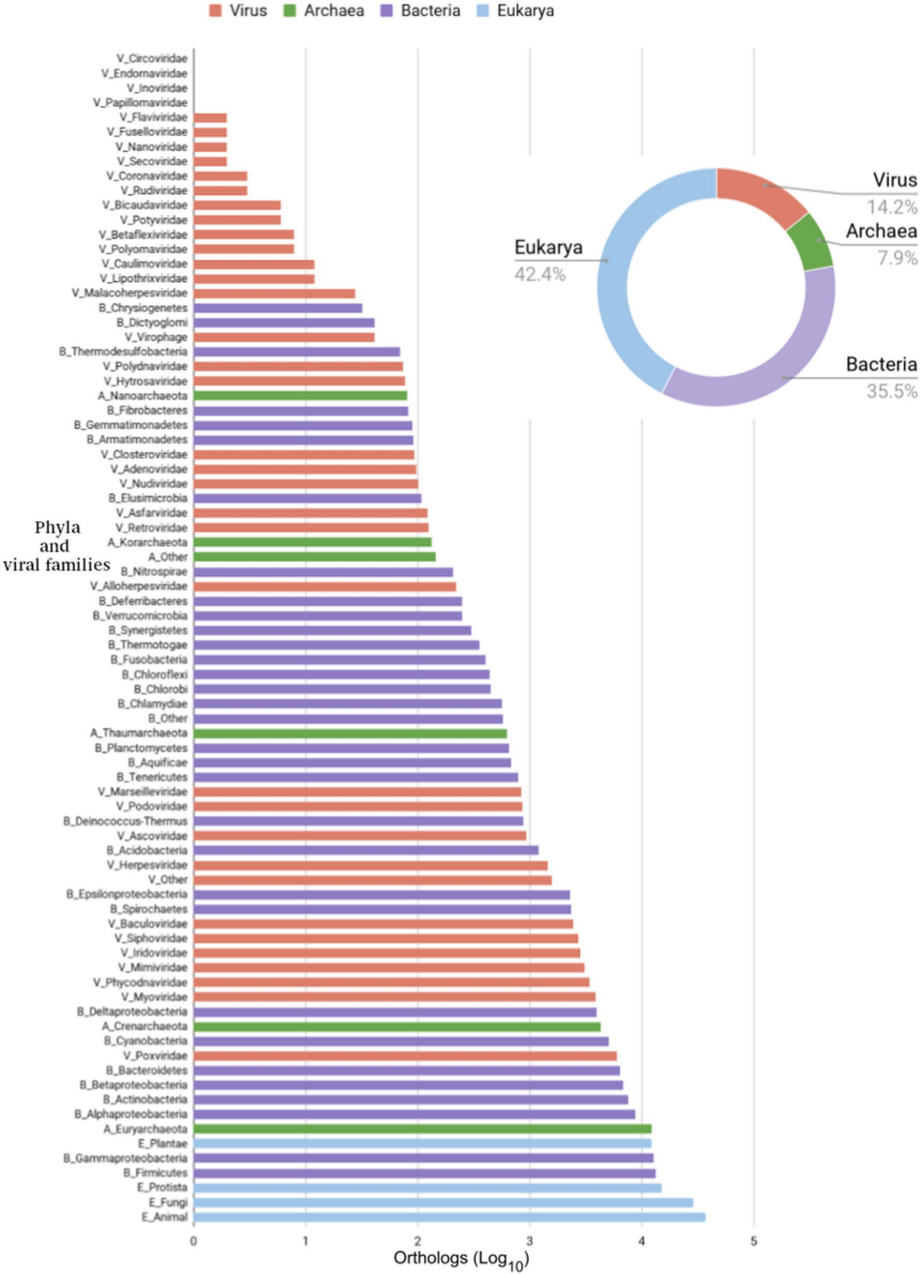
Taxonomic distribution of the orthologous clusters among NCLDVs, other non-NCLDVs, and cells. Hidden-Markov-based profiles and PSI-BLAST iterations were done using the NCLDVs orthologous clusters in order to determine cell distant homologs from KEGG database. It is observed that NCLDVs orthologous groups are shared among 40% of those eukaryotes such as animals, fungi, protists, and plants

The NCLDVs orthologs with a Pfam identifier were grouped according to their distribution in other viral groups (V) and in the major domains of life (A, Archaea; B, Bacteria; and E, Eukarya) in the Pfam database with the corresponding COG function. As shown in Fig. 3a, the orthologous clusters with unknown (COG S) and hypothetical functions (COG R) are predominantly found in the pangenome of each NCLDV family. The orthologous clusters involved in genetic information processes, including post-translational modification (COG O), transcription (COG K), replication (COG L), and nucleotide metabolism (COG F), are moderately distributed in viruses, but present in the three major domains of life. The orthologous clusters found in *Poxviridae, Phycodnaviridae*, and *Mimiviridae* with functions associated to carbohydrate (COG G), lipid (COG I), and amino acid (COG E) metabolism; coenzyme transport (COG H); signal transduction (COG T), and translation processes (COG J) are scarcely distributed at VABE. These three families have few orthologous sequences involved in signal transduction (COG T), defense mechanisms (COG V), and capsid/membrane (COG Vc) (horizontally transferred) functions, which are also present in eukaryotic hosts. No cellular structural traits like cytoskeleton (COG Z), nuclear structure (COG Y), or cell motility (COG N) were found in NCLDVs clusters. The 70% of NCLDVs pangenomic orthologous sequences have uncharacterized functions, and, at primary structure level, no host cell counterparts can be recognized. The remaining NCLDVs orthologous sequences are related to known informational, cellular, metabolic, or diverse functions, and are homologous to other viral (V), prokaryotic (P), and/or eukaryotic (E) sequences (Fig. 3b). A total of 155 Pfam orthologous clusters out of 498 from *Ascoviridae*; *Asfarviridae,* 84/203; *Iridoviridae,* 374/1723; *Marseilleviridae*, 247/3467; *Mimiviridae,* 721/2784; *Phycodnaviridae,* 1117/5853; and *Poxviridae*, 921/2461 were selected to search for cellular orthologous in the KEGG Database. Orthologous clusters with uncharacterized functions (R and S) were not included because of the apparent absence of cellular homologs at primary structure level. All cellular and viral orthologs identified by NCLDVs protein similarity were counted and classified according to viral families, prokaryotic phyla, or eukaryotic kingdoms (Fig. 4). Over 85% of cellular homologs were detected, mainly from animals, fungi, protists, Firmicutes, Gamma-proteobacteria, and plants (in that order).

### NCLDV Oxygen-Dependent Database and Viral-Cell Phylogenetic Trees

Once the NCLDV pangenomic database was built, all oxygen-dependent enzyme information and sequences were extracted. A total of 94 oxygen-dependent enzyme pangenomic clusters were identified in the NCLDV families, which can be classified into 12 protein families and one superfamily (Table 2). However, with a few exceptions, their presence and distribution within the NCLDVs families are mostly scattered. The small subunit of the ribonucleotide reductase (PF00268), which belongs to the ferritin Pfam clan (CL0044), was identified in the core of all families except for the *Poxviridae* (shell) and the *Ascoviridae* (cloud). The sulfhydryl oxidase family Erv1/Alr (PF04777) was also identified in all the NCLDVs families, in the core of Asco, Asfar, and *Marseilleviridae*, and in the shell of the remaining families. It is noteworthy that in the case of the *Poxviridae*, the sulfhydryl oxidase belongs to a different oxygen-dependent Pfam family (PF04805). Finally, three different enzymes belong to the Pfam clan Cupin CL00029: PF13532 is present in the core of the *Marseilleviridae*; PF13640 is identified in the shell of the *Mimi*- and *Phycodnaviridae*; and PF13759 present only in the *Phycodnaviridae* shell*.*

**Table 2:** O_2_-dependent enzyme domains found in pangenomic NCLDVs clusters

Family	Cofactor	Pfam ID	Pfam clan	Asco	Asfar	Irido	Marseille	Mimi	Phycodna	Pox
R2 subunit ribonucleotide reductase type Ia (R2RnR)	Fe	PF00268	CL0044	Cloud	Core	Core	Core	Core	Core	Shell
Erv1/Alr family	FAD	PF04777	–	Core	Core	Shell	Core	Shell	Shell	Shell
2OG-Fe(II) oxygenase superfamily (2OGX)	Fe(II)	PF13532 PF13640 (only mimi, phycodna) PF13759 (only phyco)	CL0029	x	x	x	Core	Shell	Shell	x
Cu/Zn superoxide dismutase	Cu/Zn	PF00080	–	x	x	x	x	Shell	Shell	Shell
Fatty acid desaturase	Fe	PF00487	CL0713	Cloud	x	x	x	x	Cloud	x
Fatty acid hydroxylase superfamily	–	PF04116	CL0713	x	x	x	x	Cloud	Cloud	x
Aspartyl/Asparaginyl beta-hydroxylase	–	PF05118	CL0029	x	x	x	x	Cloud	Shell	x
Cysteine dioxygenase	Fe(II)	PF05995	CL0029	x	x	x	x	Cloud	x	x
phytanoyl-CoA dioxygenase	Fe(II)	PF05721	CL0029	x	x	x	x	x	Cloud	x
Lytic polysaccharide mono-oxygenase	–	PF03067	CL159	x	x	x	x	x	Shell	Shell
Cytochrome b5-like heme/steroid-binding domain	Heme	PF00173	–	x	x	x	x	Cloud	x	x
Pheophorbide A oxygenase	Fe	PF08417	CL0209	x	x	x	x	x	Shell	x
Putative lipoxygenase	Fe		–	x	x	x	x	Cloud	x	x

The next step was to build phylogenetic trees for each of these oxygen-dependent enzymes, for which we selected five representative orthologous clusters for the core and shell present in most of the NCLDV pangenomes, each of which was used in the search for homologous viral and cellular sequences (cluster numbers are in the Supplementary Material).

**Fig. 5:**
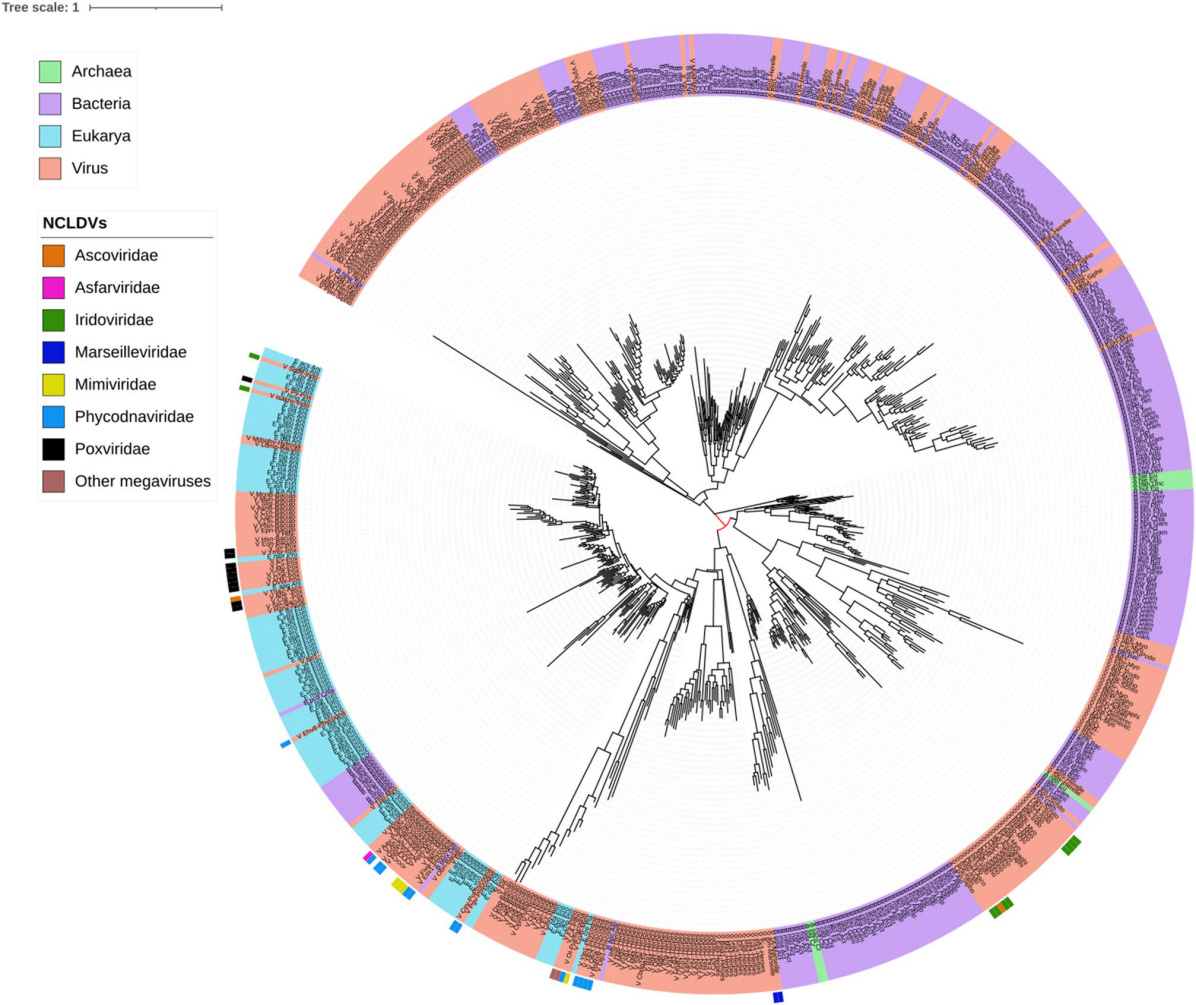
Phylogenetic tree of the small subunit of ribonucleotide reductase core cluster 165 from *Iridoviridae* (hypothetical protein/PF00268/ferritin). Taxonomic groups are written according to the color of their corresponding Domain. Each OTU is represented with a letter at the beginning according to the domain (B, Bacteria; A, Archaea y E, Eukarya) or virus (V), to the *KEGG* code, and to the taxonomic level. The phylogeny was inferred by using the most general amino acid non-reversible model with a rootstrap support of 43% (root test ID = 1) and an ultrafast bootstrap with 1000 replicates (Naser-Khdour et al. 2022)

As discussed below, all the NCLDVs are endowed with class IA ribonucleotide reductases, which are also present in numerous dsDNA bacteriophages, eukaryotes, a large number of Bacteria, and a few Archaea. In our tree (Fig. 5), most of the NCLDVs are found in a large branch which includes all the eukaryotes and some bacteria. The NCLDVs families are nevertheless scattered throughout this clade, usually in close association with their hosts. For instance, the poxviruses are interspersed in the animal clade; phycodnaviruses are found in several different branches close to fungi, protists, and plants; mimiviruses are located in two different branches, one of them close to animals, whereas the other is between several protists; and the marseillevirus branch is located at the root of this large eukaryotic clade. On the other hand, most of the asco- and iridoviral sequences are located in a branch that groups many bacteriophages of the *Myoviridae* and *Siphoviridae* families, as well as several Alpha-proteobacteria.

**Fig. 6:**
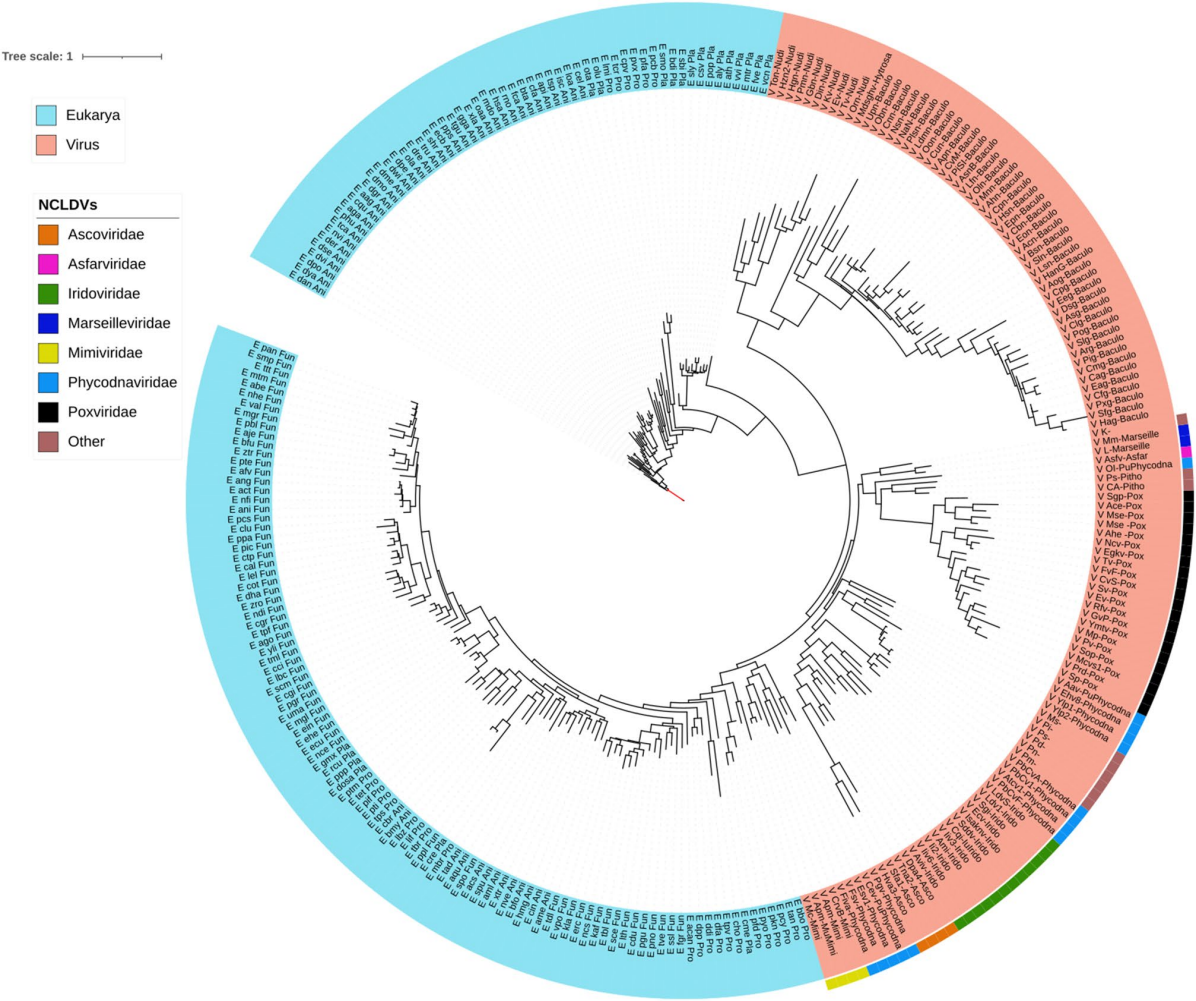
Maximum-likelihood phylogenetic tree of the Erv1/Alr core cluster 2545 from *Phycodnaviridae* (ERV1-ALR_family_protein/PF04777). Taxonomic groups are written according to the color of their corresponding domains. Each OTU is represented with a letter at the beginning according to the domain (B, Bacteria; A, Archaea y E, Eukarya) or virus (V), to the *KEGG* code, and to the taxonomic level. The phylogeny was inferred by using the most general amino acid non-reversible model with a rootstrap support of 99.4% (root test ID = 1) and an ultrafast bootstrap with 1000 replicates (Naser-Khdour et al. 2022)

The Erv/Alr tree (Fig. 6) shows that all viral sequences form a single clade which stems from one of the eukaryotic branches. Within this viral clade, the NCLDVs families are located in different branches. Two phycodnaviruses, the marseilleiviruses, and the asco-iridoviruses, as well as several poxviral proteins, form one single branch, whereas most of the phycodnavirales and the mimiviral proteins are located in a highly divergent branch along with the nudiviruses and the baculoviruses.

**Fig. 7:**
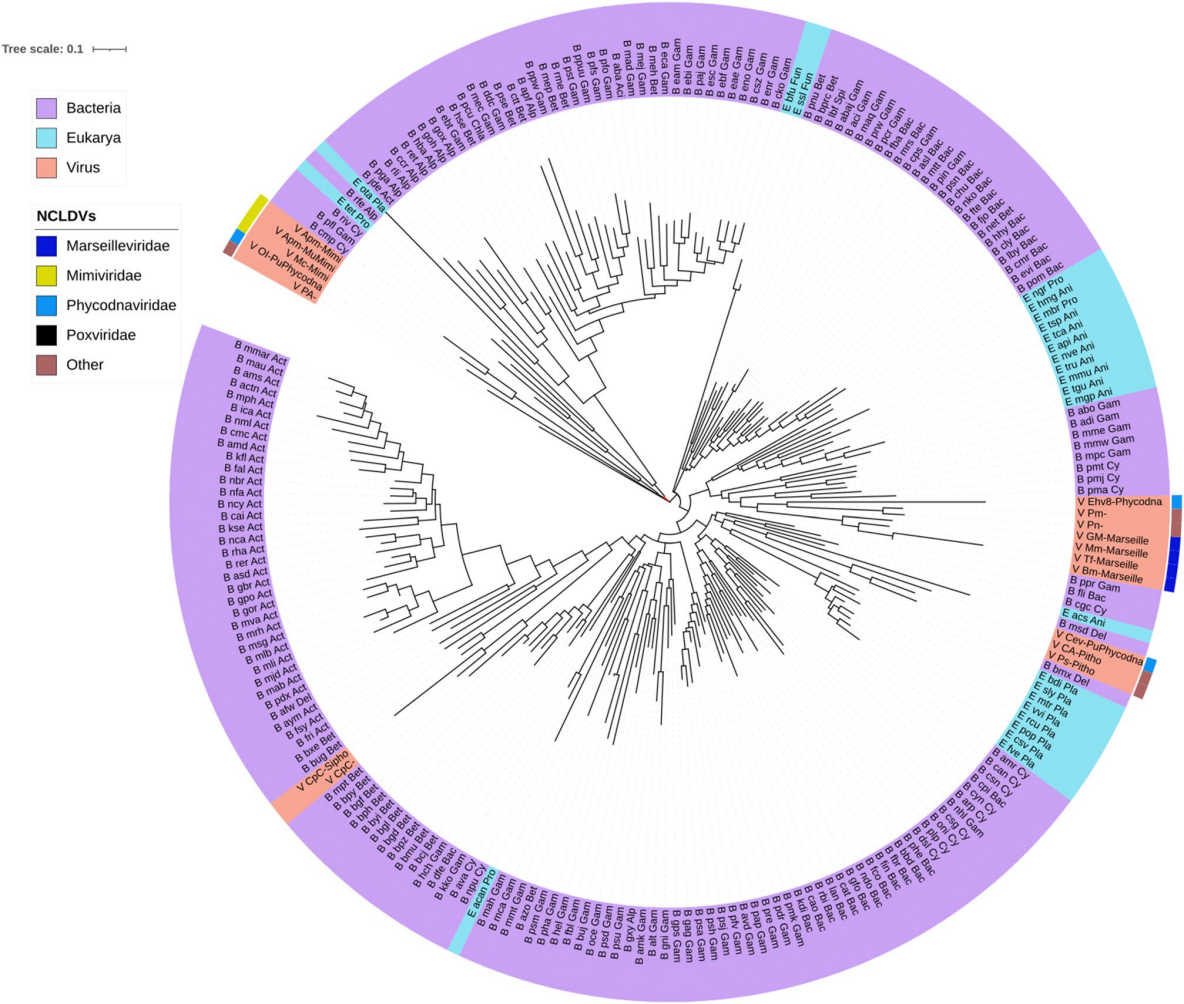
Maximum-likelihood phylogenetic tree of 2OG-Fe(II) oxygenase core cluster 662 of *Marseillevirida*e (putative-alkylated DNA repair protein/PF13532/cupin). Taxonomic groups are written according to the color of their corresponding domains. Each OTU is represented with a letter at the beginning according to the domain (B, Bacteria; A, Archaea y E, Eukarya) or virus (V), to the *KEGG* code, and to the taxonomic level. The phylogeny was inferred by using the most general amino acid non-reversible model with a rootstrap support of 35.1% (root test ID = 1) and an ultrafast bootstrap with 1000 replicates (Naser-Khdour et al. 2022)

The sequences of NCLDV putative-alkylated DNA repair protein (PF13532/CL00029) are found in different branches interspersed with different organisms (Fig. 7). In this phylogeny, the mimiviruses and a *Siphoviridae* sequence form a sister group to a large branch that includes Actinobacteria and Gamma-proteobacteria. The marseilleviruses are present as a sister group of some cyanobacteria and this branch is, in turn, a sister group to a clade grouping protists and animals. From this branch stems, a highly diverse clade which includes phycodnaviral and pandoviral sequences close to fungal and protists’ proteins as well as a clade comprising the sequences from plant-infecting RNA viruses of the families *Alphaflexiviridae, Betaflexiviridae, Secoviridae,* and *Closteroviridae*, which, in turn, forms a sister group to Proteobacteria. The pithoviruses and a phycodnavirus are found at a branch closer to the root of the tree with a couple of bacteroidetes.

**Fig. 8:**
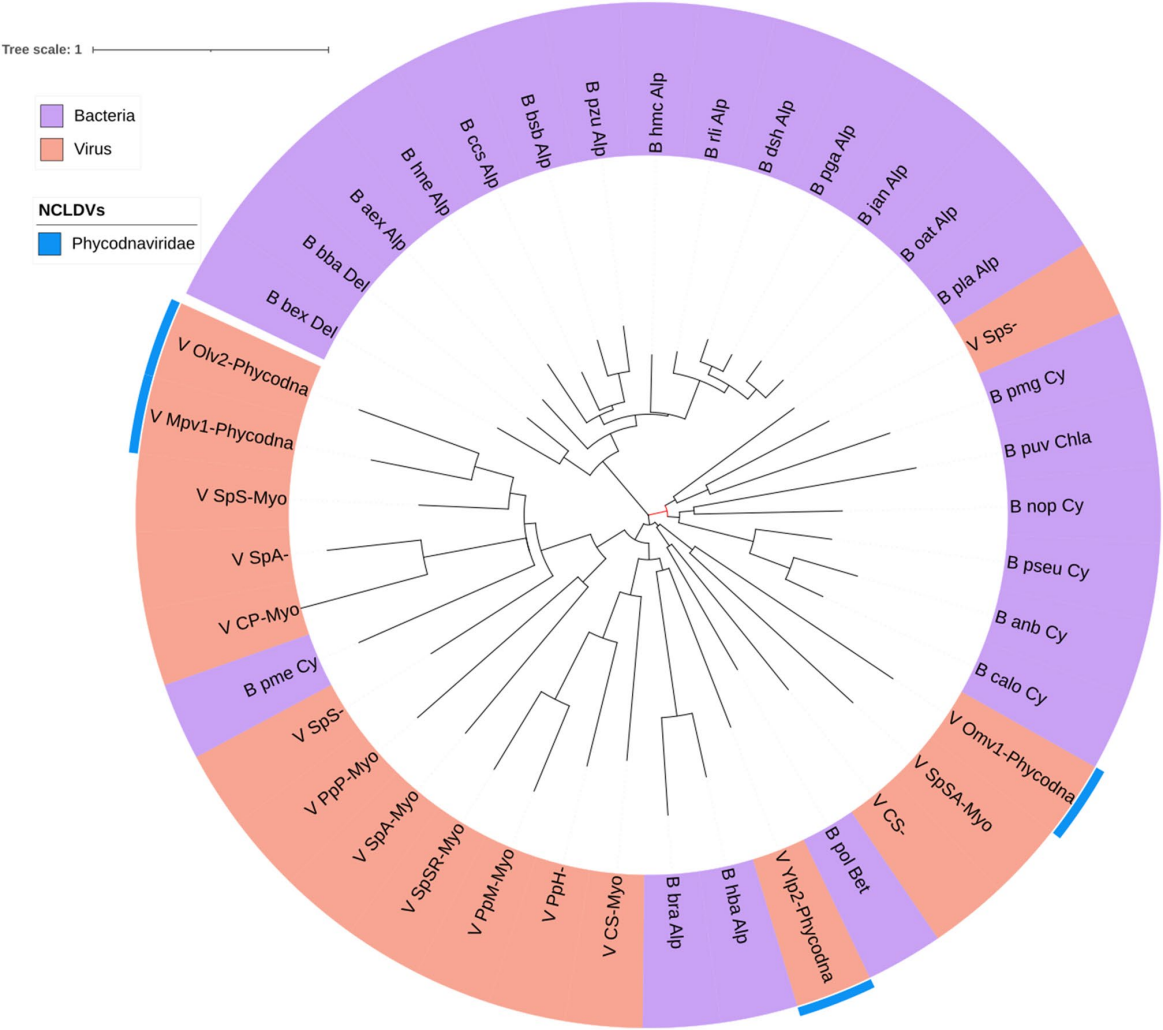
Maximum-likelihood phylogenetic-rooted tree of 2OG-Fe(II) oxygenase shell cluster 3434 of *Phycodnaviridae* (hypothetical protein/PF13759/cupin). Taxonomic groups are written according to the color of their corresponding domains. Each OTU is represented with a letter at the beginning according to the domain (B, Bacteria; A, Archaea y E, Eukarya) or virus (V), to the *KEGG* code, and to the taxonomic level. The phylogeny was inferred by using the most general amino acid non-reversible model with a rootstrap support of 43.6% (root test ID = 1) and an ultrafast bootstrap with 1000 replicates (Naser-Khdour et al. 2022)

The tree of the hypothetical protein belonging to the 2OG-FeII oxygenase PF13759/CL00029 (Fig. 8) present in the shell of the *Phycodnaviridae*, displays a bifurcation with most of the bacterial sequences on one branch and most of the viral sequences on the other. The phycodnaviral sequences are found in two different branches within the latter, which also include several myoviruses.

**Fig. 9:**
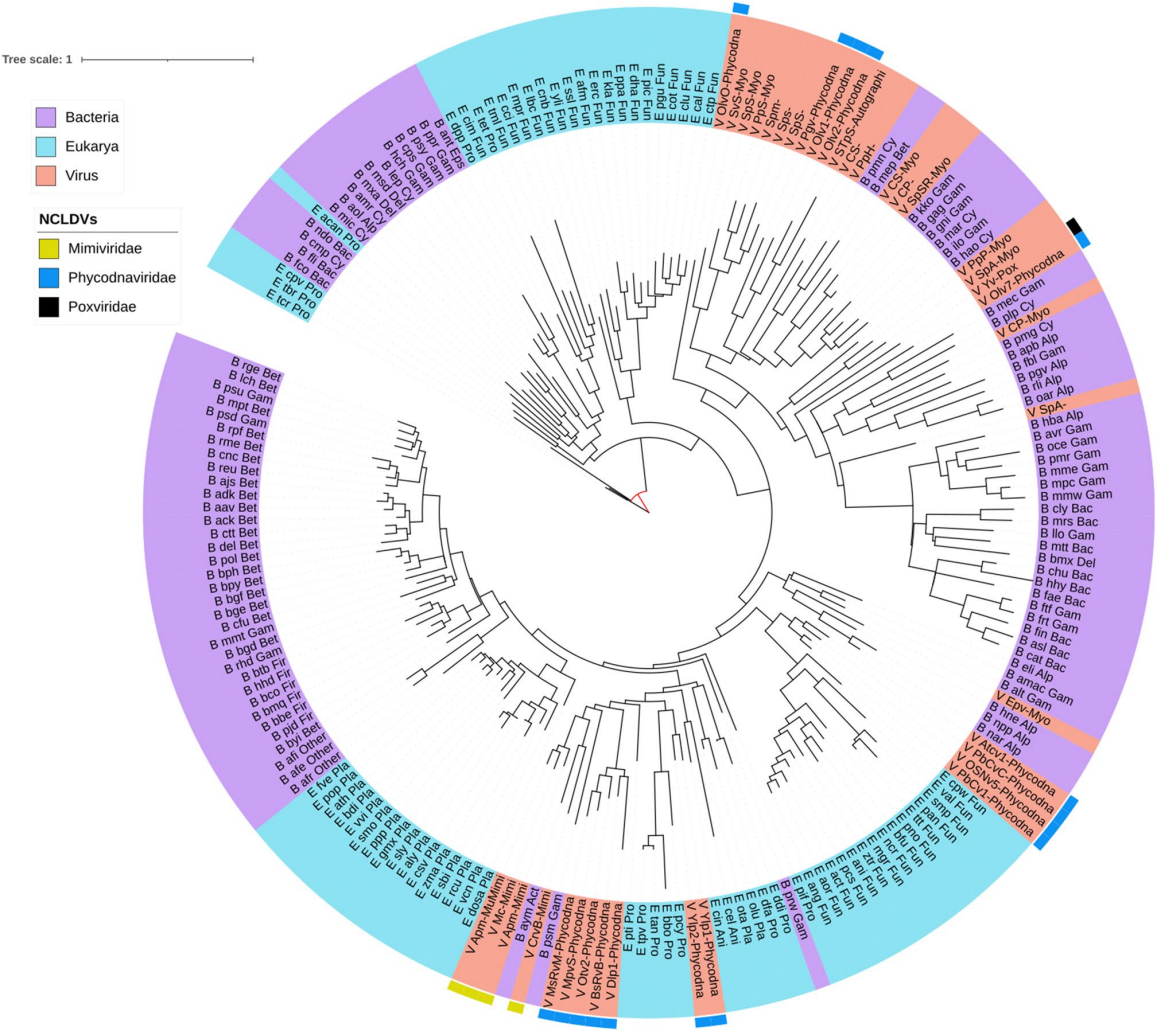
Maximum-likelihood phylogenetic-rooted tree of 2OG-Fe(II) oxygenase shell cluster 750 of *Phycodnaviridae* (hypothetical protein/PF13640/cupin). Taxonomic groups are written according to the color of their corresponding domains. Each OTU is represented with a letter at the beginning according to the domain (B, Bacteria; A, Archaea y E, Eukarya) or virus (V), to the *KEGG* code, and to the taxonomic level. The phylogeny was inferred by using the most general amino acid non-reversible model with a rootstrap support of 90.3% (root test ID = 1) and an ultrafast bootstrap with 1000 replicates (Naser-Khdour et al. 2022)

Finally, in the tree corresponding to the hypothetical protein PF13640/CL00029 (Fig. 9), the NCLDVs sequences are found in two different branches. Sequences of mimiviruses and many phycodnaviruses are located as a sister group of protists and fungi in one of the branches, whereas four of the phycodnaviral sequences are found within the branch encompassing various myoviruses and some Gamma-proteobacteria and cyanobacteria.

## Discussion and Conclusions

### Generalities of NCLDVs Pangenomes

All cellular and viral genomes have a mosaic nature due to the different origins of their sequences. In all cases, however, the highly conserved hallmark genes constitute a core that can provide key information about the nature of the ancestral entities (Lazcano et al. 1992; Delaye et al. 2005; Becerra et al. 2007; Koonin et al. 2022). NCLDVs pangenomes, i.e., the entire gene set for all viral species within each family (*Ascoviridae, Asfarviridae, Iridoviridae, Marseilleviridae, Mimiviridae, Phycodnaviridae*, and *Poxviridae*), and the phylogenetic analyses of the core clusters of proteins, provide important evolutionary data about the possible origins of these viral entities. At the most highly conserved level of the pangenome, the most conserved sequences present in all viral strains within the same family are found in the core (Fig. 1). At the next level of conservation, the shell proteins are shared by less than 90% of all viral species of the same family. Finally, the cloud comprises the viral species-specific proteins, most of which have an unknown or predicted function, and includes sequences that might have diverged so rapidly that their putative cellular homologs cannot be identified. Given the consistent biases in the viral proteome samples, a word of caution is essential when discussing these results.

The global repertoire analysis shows that NCLDVs have a rather large open pangenome (Fig. S2b), with a higher number of paralog clusters in the shell and the cloud (Table 1 and Fig. S2b). The COG Triangles pangenomic matrix partition into the shell, cloud, and core clusters was plotted for each viral family (Fig. 1). The largest cores are found on viruses that infect Acanthamoeba spp., while viruses that infect algae, invertebrates, and vertebrates exhibit the smallest core clusters. Not surprisingly, all viral proteome sequence permutations revealed that the number of core proteins decreased with the addition of a new proteome for each viral family. However, the extrapolation of each curve shows that the number of core proteins remains constant regardless of adding more proteomes (Fig. S2a). For instance, the minimum core of Poxviridae (51 proteomes) reached an asymptotic curve of four orthologous clusters (Table 1 and Fig. S2a). Analysis of all permutations also indicates that the number of shell and cloud proteins increases with the addition of proteomes. A decaying exponential model fits the estimation of this global protein repertoire. There is a huge bias between the size of the sample of genomes analyzed and the different circular areas of each compartment; this is particularly true of the core, which tends to be small compared to the others since it only includes highly conserved proteins shared with all genomes of a viral family. However, it is noteworthy that *Asfarviridae* is the only family that has a core proportionally larger than the other pangenomic compartments. There are three possible explanations for this observation: 1) there are few genomes for the family, 2) no effort has been made to discover other genera, or 3) it is simply a family with a naturally not-dynamic closed pangenome, that is, there is not a vast repertoire of new genes in the shell or cloud.

Figures 2 and 3 display the non-random distribution of the NCLDVs orthologous clusters. Genetic information clusters are found mostly in cells (prokaryotes and eukaryotes) and viruses, while uncharacterized proteins (most of which are orphan sequences) tend to be found in viruses. As summarized in Fig. 3a, in our sample, all viral proteins associated with replication, transcription, recombination, or repair functions have cellular homologs. These are, in fact, the molecular traits that define the main properties of viral replicons. The set of clusters with most cellular homologs is COG O (post-translational modification, protein turnover, chaperones). On the contrary, protein sequences with no cellular homologous and with specific (e.g., capsid formation) or unknown functions might be orphans or are simply highly diverged sequences that lost their phylogenetic imprint. Therefore, there are two possibilities: either viruses provided some core sequences to cell genomes, or viruses acquired them from cells millions of years ago. We favor the latter possibility.

The orthologous clusters shared among NCLDVs are also found in other viruses (14.2%), in Bacteria (35.5%), Archaea (7.9%) and, mostly, in Eukarya (42.4%) (Fig. 4). Regardless of their poly- or monophyletic origin, the results reported here suggest that the NCLDVs orthologous-protein clusters distributed at the core, shell, and cloud, and which are also present in the three domains of life, have an ultimate cellular origin consistent with a mechanism of host-escaping genes that may be the most plausible explanation for the origin of the core sequences (including those encoding oxygen-dependent enzymes, as discussed below) followed by a polyphyletic late accretion of a wide repertoire of cellular genes. Although it has been argued by some that viruses preceded cells, the fact that all of them are obligate intracellular entities appears to rule out this possibility. Therefore, the most likely explanation is that the homology and taxonomic distribution of the core proteins suggest they have been recruited from cellular hosts.

### Oxygen-Dependent Enzymes in NCLDVs

#### NCLDV R2 Subunit Ribonucleotide Reductase Type IA

The key role of ribonucleotide reductases (RnRs) in the early evolution of life may have been their participation in the biosynthesis of deoxyribonucleotides in the transition from RNA to DNA cellular genomes (Lazcano et al. 1988). RNRs mediate the reduction of purine- and pyrimidine ribonucleotides diphosphates to their corresponding deoxyribonucleotides by removing the -OH group on the ribose. The three different RnRs classes which catalyze this highly conserved free radical chemical reaction in the three domains of life are well understood (Torrents et al. 2002). Class I RnRs have a di-iron center that generates a tyrosyl-free radical in the presence of oxygen. These reductases have been subdivided into three types, which differ on the metal center involved in the formation of the tyrosyl radical: class IA [Fe(III)-O-Fe(III)], class IB [Mn(III)-O-Mn (III), and Fe(III)-O-Fe(III)], and class IC (Mn(IV)-O-Fe(III)]. The class IA RnRs are distributed in eukaryotes, prokaryotes, and many viruses, whereas class IB and IC RnRs are present only in bacteria. Class II RnRs require an S-adenosylcobalamin cofactor to generate the free cysteinyl radical under aerobic or anaerobic conditions, and are present in prokaryotes and bacteriophages. Class III RnRs generate a glycine radical when S-adenosyl methionine interacts with an iron-sulfur center present in prokaryotes and bacteriophages in anaerobic conditions (Torrents 2014).

Our results show that all NCLDV families described so far are endowed with the strictly aerobic class IA RnR. This holoenzyme comprises two homodimeric subunits denoted as the large subunit R1 and the small subunit R2. The R1 subunit possesses both the allosteric-binding site and the nucleotide-reduction active site, while the R2 subunit harbors the di-ferric center and the tyrosyl radical. Both motifs are easily recognized in the sequences described in our pangenomic results; however, the R2 subunit is the one that requires the presence of an oxygen atom to start the enzymatic reaction where the radical electron is transferred from the R2 tyrosine to the R1 cysteine to produce a thiol radical. The R2 subunit of class IA RnR (R2RnR) is found in all seven NCLDV families and clusterized into the pangenomic core (*Asfarviridae, Iridoviridae, Marseilleviridae, Mimiviridae, and Phycodnaviridae*), shell (*Poxviridae*), and cloud (*Ascoviridae*) (Table 2). NCLDV R2RnR sequences were grouped into the *Ribonuc_red_sm* (PF00268) Pfam family, which has a ferritin fold (CL0044) and comprises a distinctive four helical bundle with either a Fe or Mn dimer at the center (Murzin and Chothia 1992). This ferritin clan is a highly conserved iron-binding protein superfamily distributed in almost all living beings and has iron-storage functions, but has also been associated with immune (Ong et al. 2005) and stress responses (Larade and Storey 2004), and host-nutrient homeostasis during virion production in NCLDV metagenomic analyses (Moniruzzaman et al. 2020).

Consensus phylogenetic trees of core genes such as superfamily II helicase, A2L-like transcription factor, RNA polymerase A subunit, RNA polymerase B subunit, mRNA capping enzyme, A32-like packaging ATPase, myristoylated envelope protein, primase helicase, DNA polymerase, and R2RnR, have been interpreted to indicate a monophyletic origin of NCLDVs (Iyer et al. 2001, 2006; Yutin et al. 2009; Koonin and Yutin 2010). This may be the case, but given the different divergence rates of these molecules in each viral system, the use of consensus trees of a set of genes or proteins may bias the description of the evolutionary history of a complete group of viruses. For instance, the R2RnR phylogenetic tree presented in this work indicates a more complex evolutionary history than the monophyletic pattern mentioned above (Fig. 5). *Ascoviridae* and *Iridoviridae* are evolutionary related and both infect invertebrates like lepidopteran larvae (iridoviruses also infect amphibians and fish) (Federici et al. 2009), and their R2RnR phylogenetic distribution reflect two independent origins, one related to eukaryotes and the other to Alpha- and a Gamma-proteobacteria (Fig. 5.) In particular, the Gamma-proteobacteria *Francisella spp*, pathogenic bacteria of mammals and fish, have a class I RnR related to *Lymphocystis disease virus,* an iridovirus which infects fish. It has been proposed that these iridoviruses (and the new bacteriophage family *Schitoviridae* included in this study) may have acquired RnR genes from *Francisella* in a double viral/bacterial infection in an aquatic vertebrate (Lundin et al. 2010). Koonin et al. have suggested that the evolution of both R1 and R2 RnR subunits involves multiple acquisitions, losses and displacements (Yutin and Koonin 2012), and also, some lineage-specific genes in NCLDVs may have been acquired from possibly bacterial endosymbionts associated with protists (Koonin and Yutin 2019). Our study suggests that the presence of the strictly aerobic R2RnR subunit in the pangenomic repertoire of all NCLDVs may have been independently acquired at different times once significant amounts of free oxygen had accumulated during the middle Proterozoic terrestrial atmosphere approximately 2.5 Ga. This interpretation is consistent with the eukaryotic nature of the NCLDVs hosts (animals, algae, and other protists). In other words, the presence of the strictly aerobic class IA R2RnR can be considered as a biogeochemical marker that allows to date the origin of a key component of the NCLDVs’ core.

#### NCLDV Erv1/Alr

This family includes proteins that catalyze disulfide bond formation required for the stability and function of many eukaryotic (mainly in mitochondria) and periplasmic-bacterial proteins in an oxidizing environment. These sulfhydryl oxidase (SOX) domains have as a molecular signature a conserved C-X-X-C disulfide motif, adjacent to a flavin-adenine dinucleotide (FAD) prosthetic cofactor-binding site that allows the transfer of electrons from thiol substrate proteins to non-thiol electron acceptors such as oxygen (Vitu et al. 2006). This domain includes a four-helix bundle where the FAD cofactor is sheltered, as well as an additional single turn of helix (Vitu et al. 2006). The first studies of SOX in NCLDVs in the *Poxviridae* vaccinia virus strains reported that disulfide bridge formations play a key role in the intracellular mature virion and membrane assembly in the cytoplasm (Ichihashi 1981; Locker and Griffiths 1999). In the present study, the SOX (most of them identified as putative thiol oxidoreductases) domain was found in the pangenomic core (*Ascoviridae, Asfarviridae, Marseilleviridae*), shell (*Iridoviridae, Mimiviridae, Phycodnaviridae, and Poxviridae*), and few cloud clusters (*Ascoviridae, Iridodviridae, and Mimiviridae*) (Table 2). These pangenomic clusters were identified as oxygen-dependent enzyme families from the Erv1/Alr family (PF04777 Pfam without a clan classification). This protein family is distributed in eukaryotes and few bacterial groups such as Nostocales and Oscillatoriophycidea (Cyanobacteria), Rhizobiales, and Rhodobacterales (Alpha-proteobacteria), *Pirellula* (Planctomycetes), *Verrumicrobiaceae*, in *Euryarchaeota,* and in some dsDNA viruses. This domain was classified by (Yutin and Koonin 2012) as a NCLDV core protein and was used by them to demonstrate the monophyletic character of NCLDVs. However, given the mosaic nature of viral genomes, the evolutionary history of a single molecule does not necessarily reflect the evolutionary history of the entire viral group itself (Fig. 6). The tree shown in Fig. 6 displays two well-defined viral and eukaryal branches. Due to the distribution in eukaryotes and the absence of bacterial homologs, it appears that viral Erv1/Alr was acquired once by the NCLDV ancestor prior to the emergence of the last eukaryotic common ancestor and, later, in evolutionary-related insect-infecting *Nudiviridae* and *Baculoviridae* (Thézé et al. 2011). Proteins belonging to the same group of enzymes in these viral families had previously been reported and characterized. The Ac92 protein, another FAD-binding sulfhydryl oxidase present in *Baculoviridae,* has a complex quaternary structure not related to the NCLDV and eukaryal Erv1/Alr enzymes, due to possible involvement of the complex biphasic-infection caused by two structurally different virions forms (Hakim et al. 2011). The *Poxviridae* Erv1/Alr protein, E10R protein, is well conserved in this family, and is also associated with the morphogenesis of immature and mature virus particles (Senkevich et al. 2000).

#### NCLDV 2OG-Fe(II) Oxygenase Families

The non-heme 2-oxoglutarate (2OG) Fe(II)-dependent oxygenases (2OGXs) are widespread in bacteria and eukaryotes, and catalyze the reactions involved in the oxidation of organic substrates such as proline and lysine in procollagen using 2OG and a O_2_ molecule (Myllyharju and Kivirikko 1997). They play diverse functions in carnitine biosynthesis, collagen and fatty acid metabolism, phytanic acid metabolism (Loenarz and Schofield 2011), while also partaking in fundamental cellular processes such as chromatin and DNA modification, RNA synthesis, splicing, mRNA demethylation, tRNA and ribosome modification, and protein hydroxylation (Herr and Hausinger 2018). 2OGXs comprise a conserved double-stranded β-helix fold containing an HX[DE] dyad, and a carboxy-terminal histidine, both of which bind the Fe^2+^, and 2OG-binding sites and a C-ter site involved in substrate recognition. Loops play structural and catalytic functions and are hallmarks of the 2OGX classification (Islam et al. 2018). The 2OGXs are classified into the CL0029 Pfam clan, a set of proteins with a conserved barrel domain (cupin fold) that include germins and plant storage proteins (Dunwell 1998), and which contains all seven known 2OGXs. Three of these members, *2OG-FeII_Oxy_2* (PF13532), *2OG-FeII_Oxy_3* (PF13640), and *2OG-FeII_Oxy_5* (PF13759), were found in the core and shell of some NCLDV families in our study (Table 1).

Nine clusters of *Marseilleviridae* (both core and cloud), *Mimiviridae* (both shell and cloud), and *Phycodnaviridae* (cloud) were identified as PF13532 (Table 1 and Supplementary Material). This family includes AlkB, a dioxygenase protein that removes methyl groups from purines and pyrimidines to repair the DNA. The topology of the tree presented here indicates that the *Marseille*, *Mimi*-, and *Phycodnaviridae* (as well as other unclassified NCLDVs, e.g., *Pithovirus*, *Pandoravirus*, and *Pacmanvirus*) 2OXGs are distributed in different evolutionary clades. The *Pithovirus* and *Chrysochromulina ericina virus* 2OGXs share a common ancestor with those of a Cyanobacteria, a Bacteroidetes, an Alpha-proteobacteria, and a ciliate. *Pacmanvirus A23*, which is a newly discovered giant virus related to *Asfarviridae* and *Faustoviruses* (Andreani et al. 2017), has a 2OGX related to some obligate aerobic Delta- and Gamma-proteobacteria and an animal (although the closeness of the latter might be an artifact due to methodological issues such as the filtration by the query eukaryotic protein sequence size and RefSeq). This clade also clusters 2OGXs of *Mimiviridae* with Cyanobacteria and Gamma-proteobacteria, but also with some *Siphoviridae*. The sister clade of the latter groups *Marseilleviridae* with mostly animals and Cyano- and Gamma-proteobacteria. Finally, Pandoravirus and phycodnavirus branches are close to fungal and protists’ sequences, and all of these form a large clade with the 2OGX motifs of plant-infecting + ssRNA viruses and rhizosphere-related bacteria. This phylogeny might be interpreted to support the hypothesis that horizontal gene transfer has played a significant role in 2OGX evolution (Jia et al. 2017).

Likewise, 17 clusters classified as PF13640 (*2OG-FeII_Oxy_3*) were found only in *Mimiviridae* (cloud) and *Phycodnaviridae* (shell and cloud) (Table 1). Pfam classifies this family with other enzymes including AlkB, a DNA repair enzyme that removes methyl groups in purines and pyrimidines (Falnes et al. 2002). The phylogenetic tree shows again the evolutionary complexity of this enzyme, placing the *Mimiviridae* and *Phycodnaviridae* 2OGXs close to many eukaryotes and some bacteria, and distantly related to those of other phycodnavirus, which are grouped with *Myoviridae*, other bacteriophages, and many bacteria (Fig. 8).

#### Other Oxygen-Dependent Enzymes Present in NCLDV

There are other oxygen-dependent enzymes identified in the pangenomic levels of *Mimiviridae*, *Phycodnaviridae*, and *Poxviridae*. A particularly interesting one is the ubiquitous copper/zinc superoxide dismutase (Cu/Zn SOD), Pfam ID PF00080, an enzyme responsible for the conversion of superoxide radicals to hydrogen peroxide and molecular oxygen in cells (Schininà et al. 1989). Cu/Zn SOD is present in the pangenomic shell of *Mimiviridae*, *Phycodnaviridae,* and *Poxviridae*. The transition from anoxic, Cu/Zn-poor to Cu/Zn-rich oceans with a highly oxidizing atmosphere 1.8–0.8 Ga (Anbar 2008) may be the explanation for the emergence of transition-ion metal-binding motifs in the reaction center of several enzymes (Vigani and Murgia 2018) in aerobic bacteria, eukaryotes, and certainly, in NCLDV and other eukaryal virus (e.g., *Baculoviridae*) genomes. In non-heme oxygen-dependent enzymes like Cu/Zn SOD and dioxygenases discussed here, the presence of residues that participate in ion coordination indicates their oxygen requirement for their function. For example, it is well known that dioxygenases have a strict dependence on Fe2 + to activate oxygen to react with the substrate (Solomon et al. 2016). On the other hand, the Cu/Zn SOD is dependent of oxygen first by the specialization for limiting reactive oxygen species and by the oxygen-dependent maturation of Cu/Zn SOD that includes the incorporation of Cu and Zn atoms coordinated by six histidines and one aspartic acid (Valentine and de Freitas 1985). In both cases, these residues are conserved in viral sequences as shown in Fig. 7.

The fatty acid desaturase (FAD) (PF00487) is an enzyme that catalyzes the insertion of a double bond on fatty acids, and most of them are endoplasmic reticulum (ER) integral membrane proteins (Kaestner et al. 1989). FAD was found in the pangenomic clouds of *Ascoviridae* and *Phycodnaviridae*. FAD is classified into the integral membrane acyl-coA desaturase superfamily clan (CL0713), which is a family of di-iron-containing proteins that share four transmembrane-helice folds anchored to the ER membrane. This clan also contains fatty acid hydroxylases (PF04116), which are also found in the pangenomic clouds of *Mimiviridae* and *Phycodnaviridae*. This family includes fatty acid carotene hydroxylases, which are involved in the zeaxanthin synthesis by hydroxylating β-carotene, and sterol desaturases that dehydrogenate the C-5 bond in a sterol intermediate. Both of them contain two copies of a HXHH motif that coordinates Fe atoms and are highly conserved enzymes among eukaryotes.

Like the previously described 2OGXs, the next four enzymes also belong to the cupin fold family (CL0029). The aspartyl/asparaginyl beta-hydroxylases (AABH) are also oxygenases that catalyze oxidative reactions through 2OG- and Fe-binding motifs. They contain N-ter β strands and C-terminal helical domains. This enzyme was found in the pangenomic cloud of *Mimiviridae* and the pangenomic shell of *Phycodnaviridae*. A cysteine dioxygenase was found only in the pangenomic cloud of *Mimiviridae*. This enzyme is involved in the homeostatic regulation of steady-state cysteine levels and the oxidation of cysteine metabolites such as sulfate and taurine (Dominy et al. 2006).

The phytanoyl-CoA dioxygenase (PhyH) is a mostly-eukaryal and bacterial enzyme that catalyzes the oxidation of phytanic acid (Jansen et al. 2000) and hydroxylases 2-aminoethylphosphonic acid (McSorley et al. 2012), respectively. This enzyme is present only in the pangenomic cloud of *Phycodnaviridae*. The lytic polysaccharide mono-oxygenase (PF03067) was found in the pangenomic shell and cloud of *Phycodnaviridae* and *Poxviridae*. This protein plays important roles in cellulose and chitin formation (Folders et al. 2000) and is one of the 257 members of the Ig-like fold superfamily (CL0159), such as PKD, cadherins, fibronectin, bacterial Ig-like, and also viral tail fiber domains with important roles in cell–cell adhesion and signaling (Chen et al. 2018). The cytochrome b5-like heme/steroid-binding domain (PF00173) was found only in the pangenomic cloud of *Mimiviridae*. This family contains electron transport membrane-bound hemoproteins distributed in eukaryotes and in a few bacteria, such as the animal cytochrome b5, which is folded into the catalytic and membrane-binding site which anchors the microsomal membrane (Ozols 1989). Finally, the pheophorbide A oxygenase (PAO) domain (PF08417) is found close to the C-ter of a Rieske-2Fe-2S domain in cyanobacteria and plant proteins participating in the chlorophyll metabolism. This domain was found only in the pangenomic shell of *Phycodnaviridae.* PAO is involved in the viral degradation of the algal photosynthetic apparatus, and the heme-dependent cytochrome P450 family protein (Pruzinská et al. 2003), and clearly suggests a late Proterozoic origin for these viral proteins.

### Final Remarks

The different oxygen-dependent enzymes we have discussed play key roles in metabolism and cellular morphology of bacteria and eukaryotes. The defining domains of these enzymes are also found in NCLDVs, all of which thrive in the aerobic environments of their corresponding hosts. The distribution and the evolutionary analyses of these strictly oxygen-dependent enzymes demonstrate a complex history of possible gene recruitments and horizontal transfer from cells (either bacterial or eukaryal) to the NCLDVs, once the biogeochemical conditions of the Proterozoic changed to a permanently oxidized terrestrial environment after the Great Oxidation Event (GOE) 2.4–2.3 Ga. The presence of Fe-, Cu-, and other metal-binding protein motifs involved in oxygen attachment suggests that the viral recruitment of these enzymes did not lead them to lose their O_2_ dependency. The case of the aerobic RnR is particularly appealing. Its distribution in Eukaryotes, the complex phylogeny of its small subunit, and the closely related viral and host phylogenetic groups as seen in Fig. 1, in addition to the presence of this subunit in all megavirus families, specially in the pangenomic core, appear to indicate an ancient horizontal gene transfer event from early eukaryotes to a megavirus ancestor. Although the evolutionary history of each of these domains cannot represent the evolutionary history of an entire group such as megaviruses, the consilience of their distinct evolutionary stories may hint to the fact that these ancient domains could not have been acquired before the GOE. It is quite possible that these giant viruses have acquired oxygen-dependent enzymes at different times, nevertheless, always in recent times. At the same time, the RnR Ia might have been present in the last ancestor of eukaryotes (LECA) more than 2 billion years ago and acquired by horizontal transfer from bacteria, possibly from the facultative anaerobic phylum Bacteroidetes (Lundin et al. 2010). Moreover, a small megaviral core of genes that code for replication and capsid proteins has been identified, allowing to infer a monophyletic origin (Iyer et al. 2001). Hence, the consilience of these arguments: a megaviral highly conserved gene core (DNA polymerase, ATPase, D4 helicase, superfamily II helicase, RNA polymerase ɑ and β), the specific dependency on eukaryotic hosts, and the independent acquisition of different oxygen-dependent enzymes, allows us to infer that the smaller megaviruses (*Asfarviridae, Ascoviridae, Iridoviridae, Phycodnaviridae*, and *Poxviridae*) could have a monophyletic origin as proposed by Koonin et al. (Yutin et al. 2014) and evolved independently, through the gain and loss of genes (Filée 2015); and, at the same time, through ancient horizontal transfers, including those involving these oxygenic enzymes.

Viral evolution is a complex process. While in cells, vertical inheritance appears to be more significant than horizontal gene transfer, in viruses, the lateral acquisition of genes appears to be a major mechanism to acquire new traits. Our analysis confirms a non-random gene acquisition within the mosaic-like evolution process in NCLDVs, i.e., NCLDVs have not remained genetically isolated among them. While the most conserved genes present in the core in each family are mostly related to their host counterparts, the less conserved genes present in the shell of each family are phylogenetically related to other NCLDVs. While functions associated with core proteins play essential roles in cells, the same is not always true for the cloud repertoire. The results presented here are inconsistent with the proposal that NCLDVs represent a primordial fourth domain of life directly derived from the last common ancestor of living beings, a population that most likely thrived in an anaerobic environment. The processes described here do not address the monophyletic- or polyphyletic- origin of NCLDVs. This will be discussed in subsequent papers. However, our results indicate a key role for the capture-gene process in shaping the main features of this group of viruses. The same appears to be true for the O_2_-dependent enzymes discussed here, which may have been acquired after the NLCDVs evolved.

In other words, all viral proteins of which activity is present in genetic cellular processes (polymerases, exonucleases, etc.) originate from their hosts or highly related organisms. Proteins of which activity was only described on viruses come from cells as an exaptation or may have originated de novo on viruses. It is possible that this evolutionary pattern may not be restricted to NCLDVs but may be a major process of virus evolution. Therefore, as a diverse group, NCLDV families have heterogeneous pangenomes whose phylogenetic studies are evidence of extensive lateral gene transfer between them and host cells (Filée et al. 2008).

### Supplementary Information

Below is the link to the electronic supplementary material.

Supplementary file 1 Number of proteins per NCLDVs proteome. The viruses with a smaller proteome infect animalswhile those with a larger one only infect protists (JPG 69 KB)

Supplementary file 2 NCLDVs pangenome. The number of shared, essential, and unique genes for each viral family istraced as a function of the number of proteomes sequentially added to the sequence clustering a) Coreand b) Shell and Cloud orthologs (PDF 211 KB)

Supplementary file 3 The Circos visualization (Krzywinski et al. 2009) shows the absolute frequency of the orthologousclusters (band width) according to the Core, Shell, and Cloud repertoires and to the general functionalcategories: information storage and processing (yellow), cellular processes and signaling (green),metabolism (navy blue), miscellaneous functions (purple), unknown functions (gray), and probable viralfunctions (red). Likewise, these orthologous groups were classified according to the distribution in one ormore domains of life (ABE) and in viruses (V) marked in azure. The 70% of the orthologous groups withunknown function are mainly distributed at Shell and Cloud of the NCLDVs pangenome (black) (PNG 146 KB)

## References

[CR1] Altschul SF, Gish W, Miller W (1990). Basic local alignment search tool. J Mol Biol.

[CR2] Anbar AD (2008). Oceans. Elements and evolution. Science.

[CR3] Andreani J, Khalil JYB, Sevvana M (2017). Pacmanvirus, a new giant icosahedral virus at the crossroads between Asfarviridae and Faustoviruses. J Virol.

[CR4] Asgari S, Bideshi DK, Bigot Y (2017). ICTV virus taxonomy profile: Ascoviridae. J Gen Virol.

[CR5] Bäckström D, Yutin N, Jørgensen SL (2019). Virus genomes from deep sea sediments expand the ocean megavirome and support independent origins of viral gigantism. MBio.

[CR6] Becerra A, Delaye L, Islas S, Lazcano A (2007). The Very Early Stages of Biological Evolution and the Nature of the Last Common Ancestor of the Three Major Cell Domains.

[CR7] Boyer M, Madoui M-A, Gimenez G (2010). Phylogenetic and phyletic studies of informational genes in genomes highlight existence of a 4 domain of life including giant viruses. PLoS ONE.

[CR8] Capella-Gutiérrez S, Silla-Martínez JM, Gabaldón T (2009). trimAl: a tool for automated alignment trimming in large-scale phylogenetic analyses. Bioinformatics.

[CR9] Chen J, Wang B, Wu Y (2018). Structural characterization and function prediction of immunoglobulin-like fold in cell adhesion and cell signaling. J Chem Inf Model.

[CR10] Chinchar VG, Hyatt AD (2008) Iridoviruses: general features. Encyclopedia of Virology. Pp. 167–174

[CR11] Claverie J-M, Abergel C (2013). Open questions about giant viruses. Adv Virus Res.

[CR12] Contreras-Moreira B, Vinuesa P (2013). GET_HOMOLOGUES, a versatile software package for scalable and robust microbial pangenome analysis. Appl Environ Microbiol.

[CR13] Delaye L, Becerra A, Lazcano A (2005). The last common ancestor: what’s in a name?. Orig Life Evol Biosph.

[CR14] Dominy JE, Simmons CR, Karplus PA (2006). Identification and characterization of bacterial cysteine dioxygenases: a new route of cysteine degradation for eubacteria. J Bacteriol.

[CR15] Dunwell JM (1998). Cupins: a new superfamily of functionally diverse proteins that include germins and plant storage proteins. Biotechnol Genet Eng Rev.

[CR16] Eddy SR (2009). A new generation of homology search tools based on probabilistic inference. Genome Inform.

[CR17] Falnes PØ, Johansen RF, Seeberg E (2002). AlkB-mediated oxidative demethylation reverses DNA damage in Escherichia coli. Nature.

[CR18] Federici BA, Bideshi DK, Tan Y (2009). Ascoviruses: superb manipulators of apoptosis for viral replication and transmission. Curr Top Microbiol Immunol.

[CR19] Filée J (2013). Route of NCLDV evolution: the genomic accordion. Curr Opin Virol.

[CR20] Filée J (2015). Genomic comparison of closely related Giant Viruses supports an accordion-like model of evolution. Front Microbiol.

[CR21] Filée J, Pouget N, Chandler M (2008). Phylogenetic evidence for extensive lateral acquisition of cellular genes by nucleocytoplasmic large DNA viruses. BMC Evol Biol.

[CR22] Finn RD, Tate J, Mistry J (2008). The Pfam protein families database. Nucleic Acids Res.

[CR23] Folders J, Tommassen J, van Loon LC, Bitter W (2000). Identification of a chitin-binding protein secreted by *Pseudomonas aeruginosa*. J Bacteriol.

[CR24] Fu L, Niu B, Zhu Z (2012). CD-HIT: accelerated for clustering the next-generation sequencing data. Bioinformatics.

[CR25] Hakim M, Mandelbaum A, Fass D (2011). Structure of a baculovirus sulfhydryl oxidase, a highly divergent member of the erv flavoenzyme family. J Virol.

[CR26] Herr CQ, Hausinger RP (2018). Amazing diversity in biochemical roles of Fe(II)/2-oxoglutarate oxygenases. Trends Biochem Sci.

[CR27] Ichihashi Y (1981). Unit Complex of vaccinia polypeptides linked by disulfide bridges. Virology.

[CR28] ICTV (2020) Virus Taxonomy: 2019 Release. In: International Committee on Taxonomy of Viruses. https://talk.ictvonline.org/taxonomy/. Accessed 20 Sep 2020

[CR29] Islam MS, Leissing TM, Chowdhury R (2018). 2-Oxoglutarate-dependent oxygenases. Annu Rev Biochem.

[CR30] Iyer LM, Aravind L, Koonin EV (2001). Common origin of four diverse families of large eukaryotic DNA viruses. J Virol.

[CR31] Iyer LM, Balaji S, Koonin EV, Aravind L (2006). Evolutionary genomics of nucleo-cytoplasmic large DNA viruses. Virus Res.

[CR32] Jansen GA, Hogenhout EM, Ferdinandusse S (2000). Human phytanoyl-CoA hydroxylase: resolution of the gene structure and the molecular basis of Refsum’s disease. Hum Mol Genet.

[CR33] Jia B, Jia X, Kim KH, Jeon CO (2017). Integrative view of 2-oxoglutarate/Fe(II)-dependent oxygenase diversity and functions in bacteria. Biochim Biophys Acta Gen Subj.

[CR34] Kaestner KH, Ntambi JM, Kelly TJ, Lane MD (1989). Differentiation-induced gene expression in 3T3-L1 preadipocytes. A second differentially expressed gene encoding stearoyl-CoA desaturase. J Biol Chem.

[CR35] Katoh K, Misawa K, Kuma K-I, Miyata T (2002). MAFFT: a novel method for rapid multiple sequence alignment based on fast Fourier transform. Nucleic Acids Res.

[CR36] Koonin EV, Yutin N (2010). Origin and evolution of eukaryotic large nucleo-cytoplasmic DNA viruses. Intervirology.

[CR37] Koonin EV, Yutin N (2012). Nucleo-cytoplasmic Large DNA Viruses (NCLDV) of Eukaryotes.

[CR38] Koonin EV, Yutin N, Kielian M, Mettenleiter TC, Roossinck MJ (2019). Chapter Five—Evolution of the large nucleocytoplasmic DNA viruses of eukaryotes and convergent origins of viral gigantism. Advances in virus research.

[CR39] Koonin EV, Senkevich TG, Dolja VV (2006). The ancient virus world and evolution of cells. Biol Direct.

[CR40] Koonin EV, Dolja VV, Krupovic M (2015). Origins and evolution of viruses of eukaryotes: The ultimate modularity. Virology.

[CR41] Koonin EV, Dolja VV, Krupovic M (2022). The logic of virus evolution. Cell Host Microbe.

[CR42] Kristensen DM, Kannan L, Coleman MK (2010). A low-polynomial algorithm for assembling clusters of orthologous groups from intergenomic symmetric best matches. Bioinformatics.

[CR43] Krzywinski M, Schein J, Birol I (2009). Circos: an information aesthetic for comparative genomics. Genome Res.

[CR44] Larade K, Storey KB (2004). Accumulation and translation of ferritin heavy chain transcripts following anoxia exposure in a marine invertebrate. J Exp Biol.

[CR45] Lazcano A, Guerrero R, Margulis L, Oró J (1988). The evolutionary transition from RNA to DNA in early cells. J Mol Evol.

[CR46] Lazcano A, Fox GE, Oró J, Mortlock R (1992). Life before DNA: The origin and evolution of early archean cells. The evolution of metabolic funcion.

[CR47] Legendre M, Arslan D, Abergel C, Claverie J-M (2012). Genomics of megavirus and the elusive fourth domain of life. Commun Integr Biol.

[CR48] Letunic I, Bork P (2016). Interactive tree of life (iTOL) v3: an online tool for the display and annotation of phylogenetic and other trees. Nucleic Acids Res.

[CR49] Locker JK, Griffiths G (1999). An unconventional role for cytoplasmic disulfide bonds in vaccinia virus proteins. J Cell Biol.

[CR50] Loenarz C, Schofield CJ (2011). Physiological and biochemical aspects of hydroxylations and demethylations catalyzed by human 2-oxoglutarate oxygenases. Trends Biochem Sci.

[CR51] Lundin D, Gribaldo S, Torrents E (2010). Ribonucleotide reduction—horizontal transfer of a required function spans all three domains. BMC Evol Biol.

[CR52] Madera M, Gough J (2002). A comparison of profile hidden Markov model procedures for remote homology detection. Nucleic Acids Res.

[CR53] McSorley FR, Wyatt PB, Martinez A (2012). PhnY and PhnZ comprise a new oxidative pathway for enzymatic cleavage of a carbon-phosphorus bond. J Am Chem Soc.

[CR54] Medini D, Donati C, Tettelin H (2005). The microbial pan-genome. Curr Opin Genet Dev.

[CR55] Moniruzzaman M, Martinez-Gutierrez CA, Weinheimer AR, Aylward FO (2020). Dynamic genome evolution and complex virocell metabolism of globally-distributed giant viruses. Nat Commun.

[CR56] Moreira D, Brochier-Armanet C (2008). Giant viruses, giant chimeras: the multiple evolutionary histories of Mimivirus genes. BMC Evol Biol.

[CR57] Murzin AG, Chothia C (1992). Protein architecture: new superfamilies. Curr Opin Struct Biol.

[CR58] Myllyharju J, Kivirikko KI (1997). Characterization of the iron- and 2-oxoglutarate-binding sites of human prolyl 4-hydroxylase. EMBO J.

[CR59] Naser-Khdour S, Quang Minh B, Lanfear R (2022). Assessing confidence in root placement on phylogenies: an empirical study using nonreversible models for mammals. Syst Biol.

[CR82] Nguyen LT, Schmidt HA, von Haeseler A, Minh BQ (2015). IQ-TREE: a fast and effective stochastic algorithm for estimating maximum-likelihood phylogenies. Mol Biol Evol.

[CR60] Ong DST, Wang L, Zhu Y (2005). The response of ferritin to LPS and acute phase of Pseudomonas infection. J Endotoxin Res.

[CR61] Ozols J (1989). Structure of cytochrome b5 and its topology in the microsomal membrane. Biochim Biophys Acta.

[CR62] Pruzinská A, Tanner G, Anders I (2003). Chlorophyll breakdown: pheophorbide a oxygenase is a Rieske-type iron-sulfur protein, encoded by the accelerated cell death 1 gene. Proc Natl Acad Sci USA.

[CR83] Raoult D, Audic S, Robert C (2004). The 1.2-megabase genome sequence of mimivirus. Science.

[CR63] Schininà ME, Barra D, Bossa F (1989). Primary structure from amino acid and cDNA sequences of two Cu, Zn superoxide dismutase variants from *Xenopus laevis*. Arch Biochem Biophys.

[CR64] Senkevich TG, White CL, Koonin EV, Moss B (2000). A viral member of the ERV1/ALR protein family participates in a cytoplasmic pathway of disulfide bond formation. Proc Natl Acad Sci USA.

[CR65] Sobhy H, Scola BL, Pagnier I (2015). Identification of giant Mimivirus protein functions using RNA interference. Front Microbiol.

[CR66] Solomon EI, Goudarzi S, Sutherlin KD (2016). O Activation by non-heme iron enzymes. Biochemistry.

[CR67] Tamames J, Gil R, Latorre A (2007). The frontier between cell and organelle: genome analysis of Candidatus *Carsonella ruddii*. BMC Evol Biol.

[CR68] Tatusov RL (1997). A genomic perspective on protein families. Science.

[CR69] Tettelin H, Masignani V, Cieslewicz MJ (2005). Genome analysis of multiple pathogenic isolates of Streptococcus agalactiae: implications for the microbial “pan-genome”. Proc Natl Acad Sci USA.

[CR70] Tettelin H, Riley D, Cattuto C, Medini D (2008). Comparative genomics: the bacterial pan-genome. Curr Opin Microbiol.

[CR71] Thézé J, Bézier A, Periquet G (2011). Paleozoic origin of insect large dsDNA viruses. Proc Natl Acad Sci U S A.

[CR72] Tidona C, Darai G (2011). The springer index of viruses.

[CR73] Torrents E (2014). Ribonucleotide reductases: essential enzymes for bacterial life. Front Cell Infect Microbiol.

[CR74] Torrents E, Aloy P, Gibert I, Rodríguez-Trelles F (2002). Ribonucleotide reductases: divergent evolution of an ancient enzyme. J Mol Evol.

[CR75] Valentine JS, de Freitas DM (1985). Copper-zinc superoxide dismutase: a unique biological “ligand” for bioinorganic studies. J Chem Educ.

[CR76] Vigani G, Murgia I (2018). Iron-requiring enzymes in the spotlight of oxygen. Trends Plant Sci.

[CR77] Vitu E, Bentzur M, Lisowsky T (2006). Gain of function in an ERV/ALR sulfhydryl oxidase by molecular engineering of the shuttle disulfide. J Mol Biol.

[CR78] Woyke T, Rubin EM (2014). Evolution. Searching for new branches on the tree of life. Science.

[CR79] Yutin N, Koonin EV (2012). Hidden evolutionary complexity of nucleo-cytoplasmic large DNA viruses of eukaryotes. Virol J.

[CR80] Yutin N, Wolf YI, Raoult D, Koonin EV (2009). Eukaryotic large nucleo-cytoplasmic DNA viruses: clusters of orthologous genes and reconstruction of viral genome evolution. Virol J.

[CR81] Yutin N, Wolf YI, Koonin EV (2014). Origin of giant viruses from smaller DNA viruses not from a fourth domain of cellular life. Virology.

