# Supplementary figures and images for: Pangenomic Analysis of Nucleo-Cytoplasmic Large DNA Viruses. I: The Phylogenetic Distribution of Conserved Oxygen-Dependent Enzymes Reveals a Capture-Gene Process
Source: J Mol Evol. 2023 Aug 1;91(5):647–68. doi: 10.1007/s00239-023-10126-z (PMC10598087; doi:10.1007/s00239-023-10126-z)

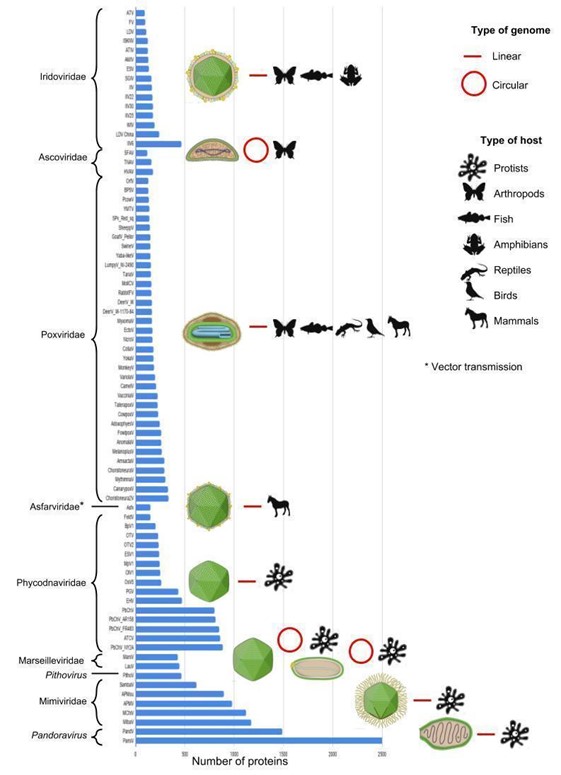

Supplement: Supplementary file 1 — Number of proteins per NCLDVs proteome. The viruses with a smaller proteome infect animalswhile those with a larger one only infect protists (JPG 69 KB) [file 239_2023_10126_MOESM1_ESM.jpg]

**Iridoviridae (n=26)**

**a) Core**

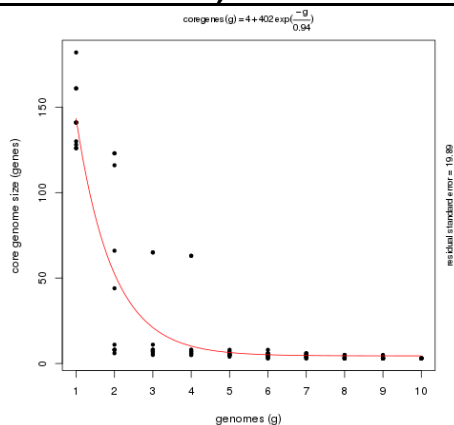

**b) Shell and Cloud**

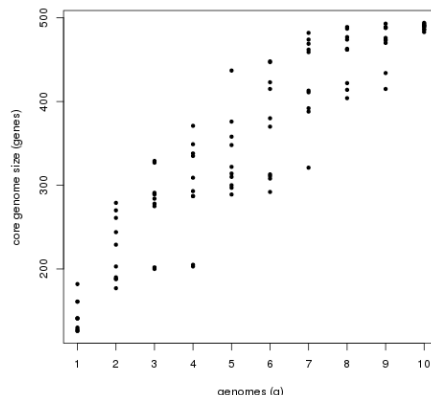

**Ascoviridae (n=6)**

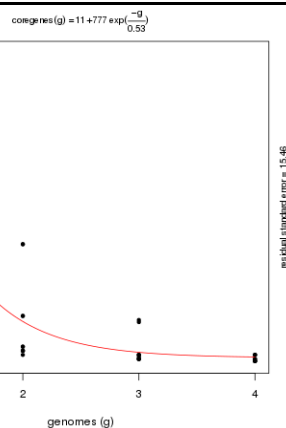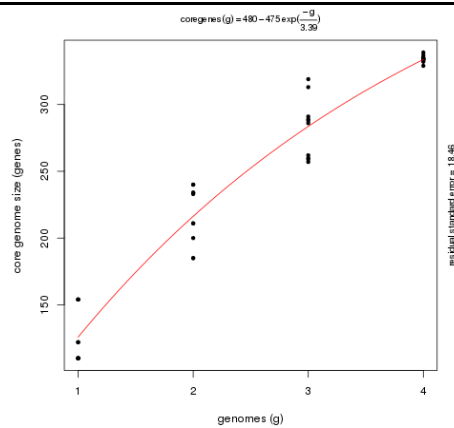

**Poxviridae (n=51)**

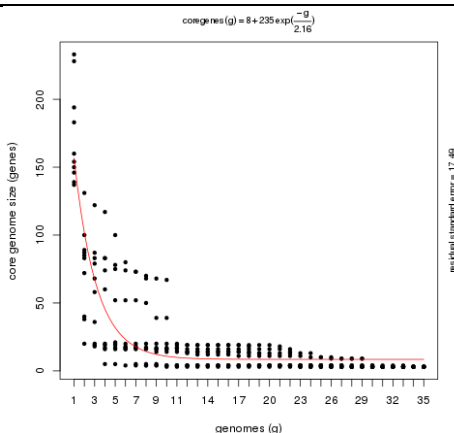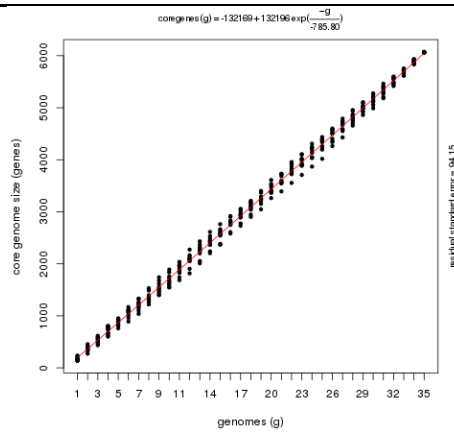

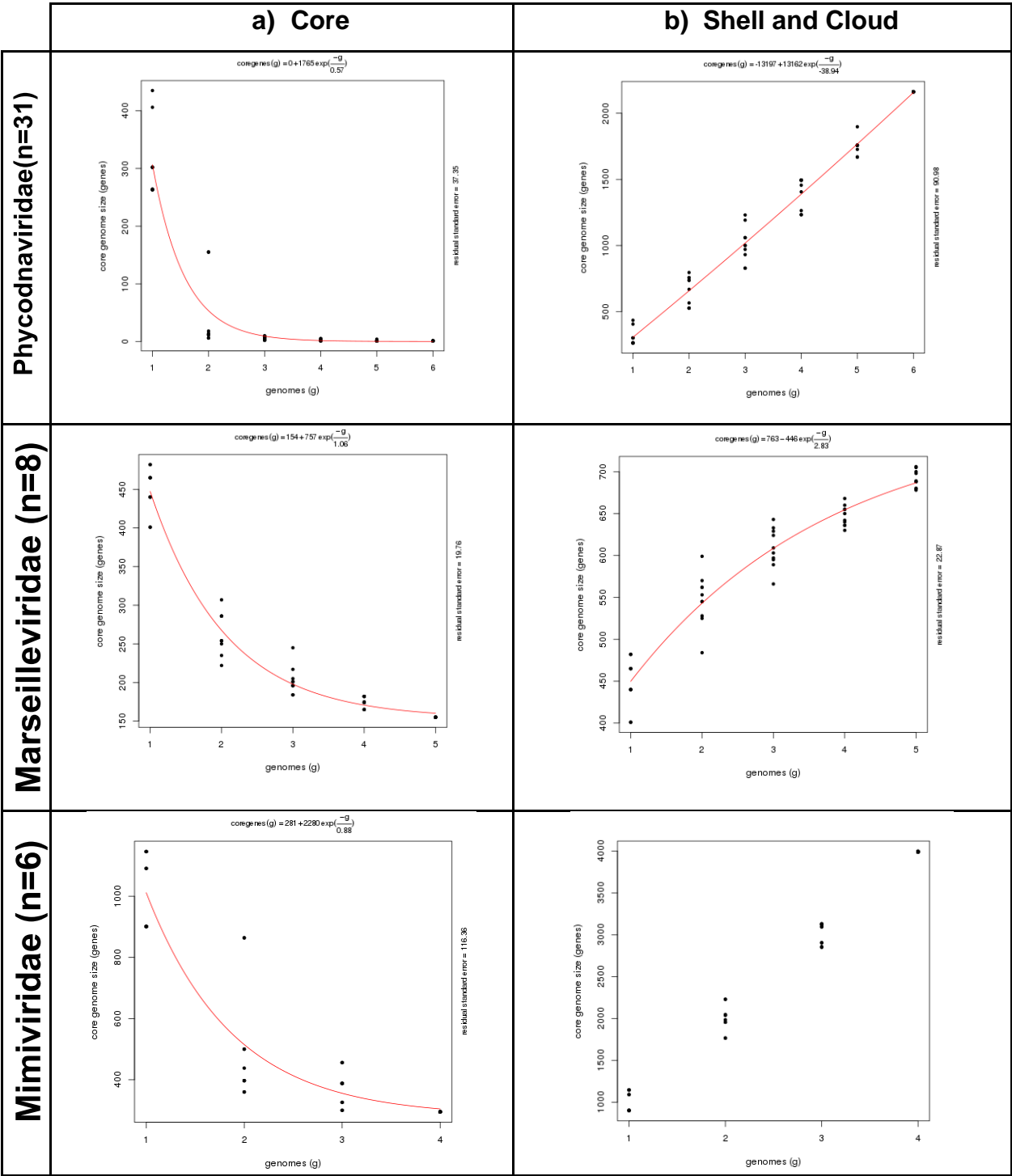

Supplement: Supplementary file 2 — NCLDVs pangenome. The number of shared, essential, and unique genes for each viral family istraced as a function of the number of proteomes sequentially added to the sequence clustering a) Coreand b) Shell and Cloud orthologs (PDF 211 KB) [file 239_2023_10126_MOESM2_ESM.pdf]

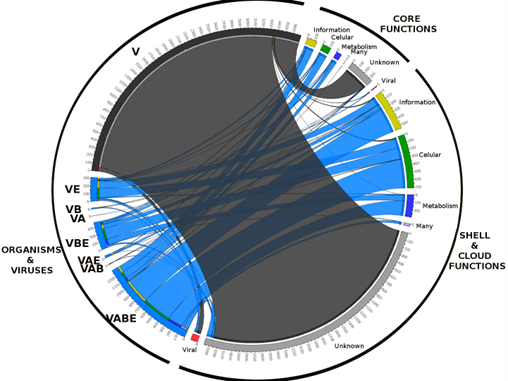

Supplement: Supplementary file 3 — The Circos visualization (Krzywinski et al. 2009) shows the absolute frequency of the orthologousclusters (band width) according to the Core, Shell, and Cloud repertoires and to the general functionalcategories: information storage and processing (yellow), cellular processes and signaling (green),metabolism (navy blue), miscellaneous functions (purple), unknown functions (gray), and probable viralfunctions (red). Likewise, these orthologous groups were classified according to the distribution in one ormore domains of life (ABE) and in viruses (V) marked in azure. The 70% of the orthologous groups withunknown function are mainly distributed at Shell and Cloud of the NCLDVs pangenome (black) (PNG 146 KB) [file 239_2023_10126_MOESM3_ESM.png]
